# Biaxial testing system for characterization of mechanical and rupture properties of small samples

**DOI:** 10.1016/j.ohx.2022.e00333

**Published:** 2022-06-28

**Authors:** Andrea Corti, Tariq Shameen, Shivang Sharma, Annalisa De Paolis, Luis Cardoso

**Affiliations:** City College of the City University of New York, New York, NY 10029, USA

**Keywords:** Material testing system, Uniaxial, Biaxial, Biomechanics, Tensile testing, Rupture Testing

## Abstract

The study of damage and rupture of soft tissues using a tensile testing system is essential to understand the limits of mechanical behavior and loss of function in diseased tissues. However, commercial material testing systems are often expensive and may not be fully suitable for rupture tests of small samples. While several research laboratories have developed custom, less expensive, uniaxial or biaxial devices, there is a need for an open source, inexpensive, accurate and easy to customize biaxial material testing system to perform rupture tests in small soft samples.

We designed a testing system (BiMaTS) that (a) was shown able to perform uniaxial and biaxial tests, (b) offers a large travel range for rupture tests of small samples, (c) maintains a centered field of view for effective strain mapping using digital image correlation, (d) provides a controlled temperature environment, (e) utilize many off-the-shelve components for easy manufacture and customization, and it is cost effective (∼$15 K).

The instrument performance was characterized using 80%-scaled down, ASTM D412-C shaped PDMS samples. Our results demonstrate the ability of this open source, customizable, low-cost, biaxial materials testing system to successfully characterize the mechanical and rupture properties of small samples with high repeatability and accuracy.

## Graphical abstract

**Figure:**
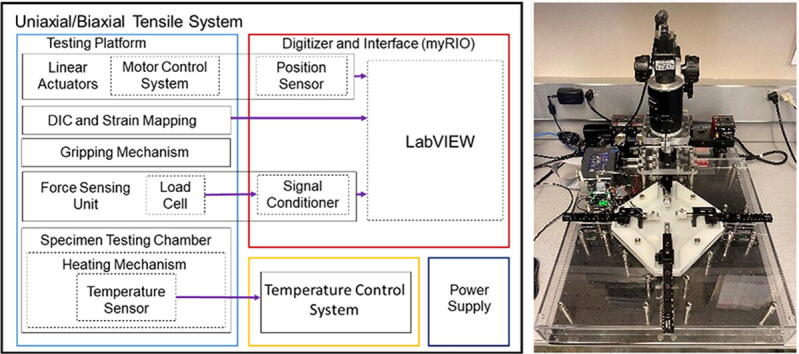

## Hardware in context

The mechanical properties of soft tissues in the body change over time due to many biological, environmental and extrinsic factors, including ageing, the onset of a pathology, or trauma. There exist several approaches to characterize the biomechanical properties of tissues ex vivo, including tensile / compression testing, indentation testing, ultrasound, atomic force microscopy, etc. In particular, the study of damage and rupture of soft tissues using a uniaxial tensile testing system [1] is essential to understand the limits of mechanical behavior and loss of function in diseased tissues [2]. However, commercial material testing systems (e.g. Tytron 250 Microforce Testing System, ElectroForce Planar Biaxial TestBench, Cell Scale BioTester [3], [4], etc.) are often expensive ($50 K-$100 K + ) and several research laboratories have developed custom, less expensive, uniaxial or biaxial devices [5], [6], [7], [8], [9], [10], [11], [12], or open source [13]. Also, most systems may not be suitable for rupture tests of small samples due to a limited travel range, their gripping mechanism [14], and/or load cell range. Thus, there is a need for an open source biaxial material testing system to perform rupture tests in soft tissues, inexpensive, accurate and easy to customize by the researcher.

In this study, we report the design of an open source, low-cost biomechanical testing system that allows us to characterize the mechanical and rupture properties of small samples with high accuracy and precision (Table 1). The proposed biaxial materials testing system (BiMaTS) comprises four high performance actuators with inline load cells (8.90 N), high resolution imaging with image-based strain measurement tools [15], [16], integrated temperature controlled media bath, and a LabView-based user interface for automated computer controlled testing with real-time feedback. The BiMaTS is able (1) to perform uniaxial and biaxial tests, (2) offers a large travel range (100 mm) for rupture tests of small samples (∼5–25 mm long), (3) maintains a centered field of view for effective strain mapping using digital image correlation (DIC), (4) provides a controlled environment for testing of biological tissues under immersion (e.g. phosphate saline solution) and physiological temperature control, and (5) utilize many off-the-shelve components for easy manufacture. The repeatability, accuracy and overall performance of the instrument (i.e. displacement, actuator velocity, force, temperature, gripping slippage, strain mapping) was characterized using Polydimethylsiloxane (PDMS) samples with ASTM D412-C standard shape, following the recommended testing guidelines for rubber-like materials.

**Table 1:** Specifications table.

Hardware name	*Biaxial Material Testing System (BiMaTS)*
Subject area	• *Engineering and Material Science*
Hardware type	• *Other: Material Testing System*
Closest commercial analog	• *ADMET eXpert 8000 Series Planar Biaxial Test Machine* • *ZwickRoell Biaxial Testing Machine* • *TA Instruments: ElectroForce Planar Biaxial TestBench Instrument* • *CellScale BioTester*
Open Source License	*CC-BY-4.0*
Cost of Hardware	*Approximate cost of hardware* $14,868.67
Source File Repository	https://doi.org/10.17632/8sbwf397jk.1

## Hardware description

**Fig. 1:**
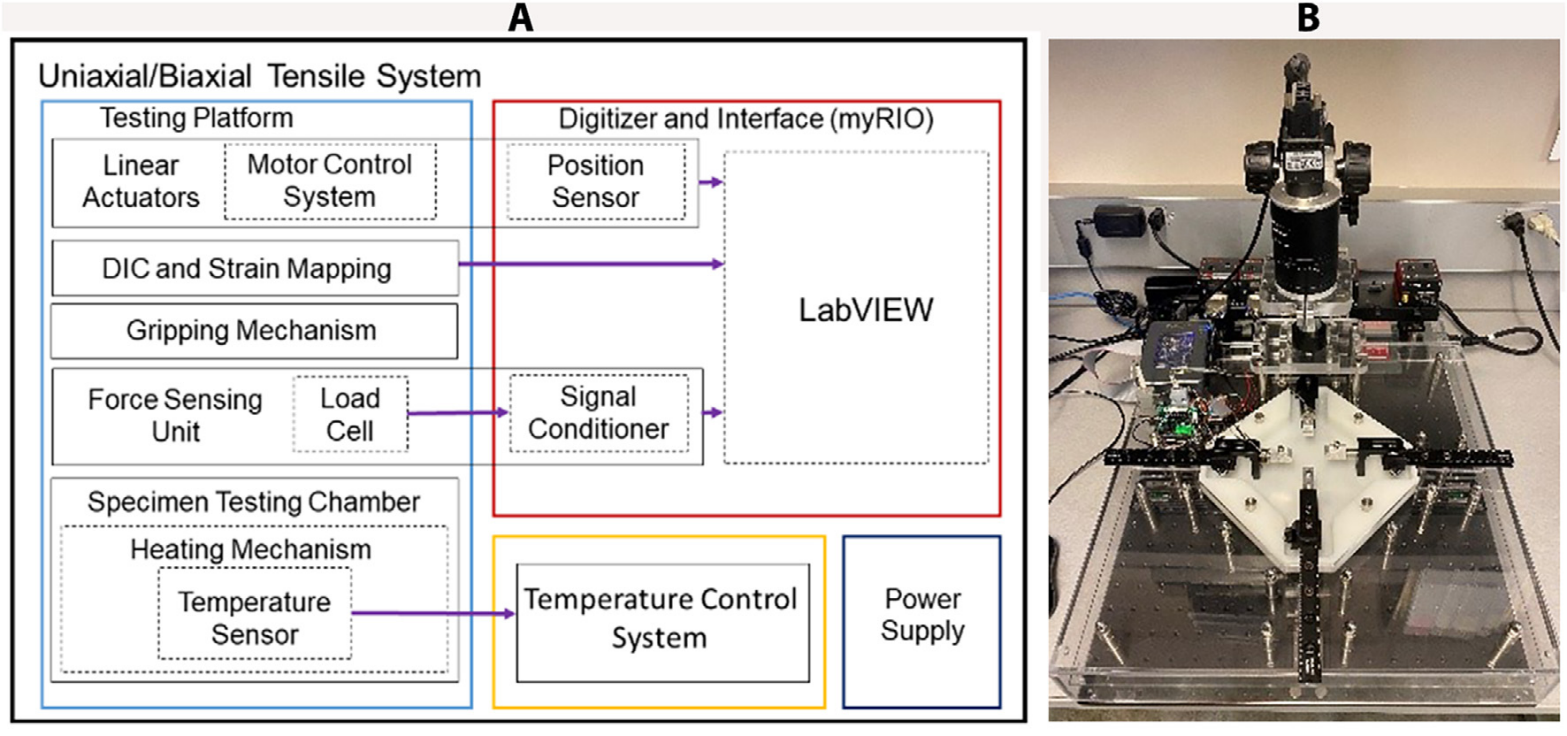
(A) Biaxial Material Testing System (BiMaTS) building blocks: testing platform; specimen testing chamber; force sensing unit; tissue gripping mechanism; optical strain mapping; and graphical user interface for control and operation of the system. (B) Photograph of the BiMaTS instrument.

The BiMaTS (Fig. 1) comprises six building blocks or subsystems: (1) testing platform; (2) specimen testing chamber; (3) force sensing unit; (4) sample gripping mechanism; (5) optical strain mapping; and (6) computer and Graphical User Interface (GUI) for control and operation of the system (Table 2).

### Testing platform

**Fig. 2:**
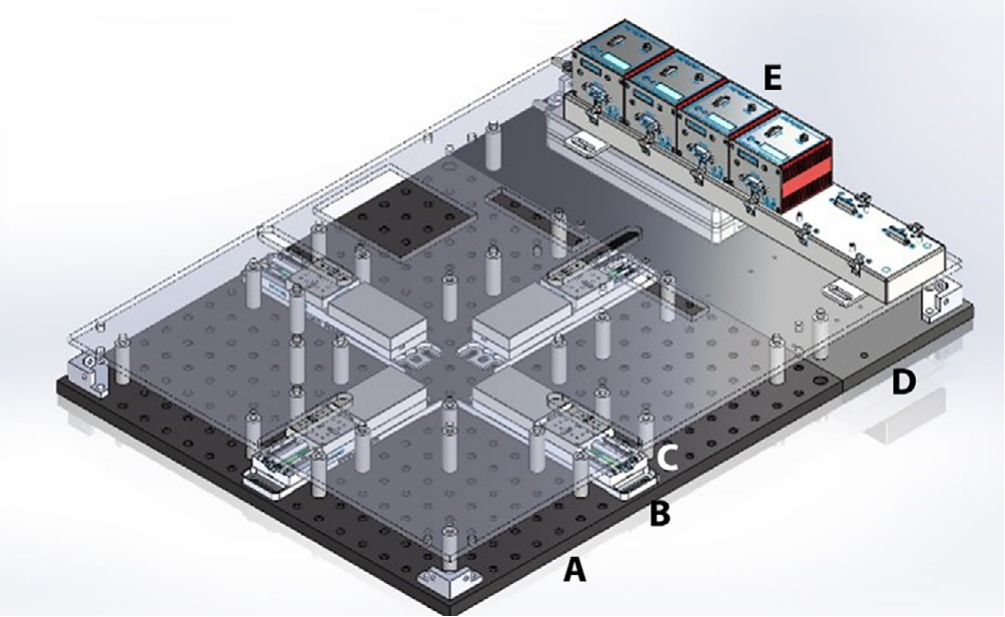
BiMaTS testing platform. (A) breadboard, (B) Breadboard Mounting Adapter (MTS50A-Z8), (C) linear actuator (MTS50-Z8), (D) back extension plate, (E) DC Servo Motor Driver (KBD101).

The testing platform consists of a rigid aluminum breadboard typically used for laser and optical applications (Fig. 2). This breadboard provides an excellent means to adjust the location of any components while maintaining excellent alignment among actuators. Four linear actuators (Thorlabs MTS50-Z8-50 mm motorized translation stage) were positioned in a cruciform configuration to allow the stretching of samples along two orthogonal axes, while maintaining the sample’s region of interest at the center of the field of view. Each linear actuator has a 50 mm range of motion with a 25 lb horizontal load capacity, 2.4 mm/s maximal velocity, and 4.5 mm/s^2^ maximal acceleration (full technical specifications are found at https://www.thorlabs.com/newgrouppage9.cfm?objectgroup_id = 3002&pn = MTS50-Z8). Therefore, two combined actuators lead to a full 100 mm range of motion in X and Y directions. DC Servo motor actuators are powered and controlled by a DC Servo Motor Driver (Thorlabs’ K-Cube Series KBD101s), which provide closed-loop feedback control via built-in Hall Effect position encoders for precise motion of the actuator (minimum achievable incremental movement of 0.05 μm, minimum repeatable incremental movement of 0.8 μm, bidirectional repeatability of 1.6 μm, backslash < 6 μm, and home location accuracy of ± 4.0 μm).

### Specimen testing chamber

**Fig. 3:**
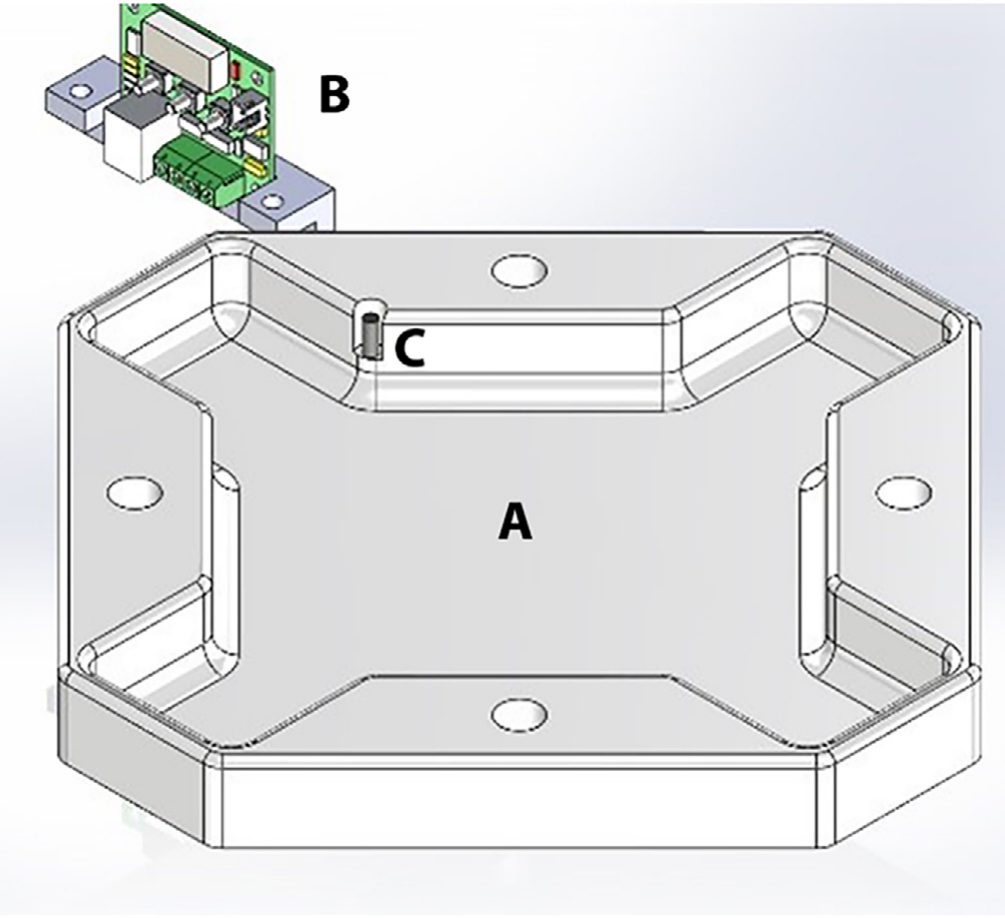
Specimen testing chamber. (A) specimen testing chamber has a cross-shaped design made of biocompatible Polytetrafluoroethylene (PTFE), (B) Temperature control board (W1209), (C) temperature sensor.

The specimen testing chamber has a cross-shaped design made of biocompatible Polytetrafluoroethylene (PTFE) and was designed for examination of samples in air and under fluid immersion (Fig. 3). To control the fluid temperature used for testing samples under immersion, the chamber has a Peltier ceramic actuator driven by a W1209 temperature control board. The Peltier plate and W1209 control board are powered up by independent power supplies. The heating side of the Peltier thermal actuator was attached to a 10 mm × 10 mm × 1 mm stainless steel plate that lies beneath the chamber's base. The W1209 reads the fluid temperature using a 4 mm diameter sensor that was placed within the chamber and determines whether the Peltier plate should be powered up or down to maintain the chamber fluid within 37 ± 1 °C. The operation settings of the W1209 temperature control circuit include the P0 parameter, which was set to ‘H' as the chamber needs to be heated. The target temperature was set to 37.0 °C, with the hysteresis parameter P1 selected at 0.1 °C. The lower limit, parameter P3, of the temperature range was configured to 35.0 °C, and the upper limit/relay off, parameter P2 and P6 were chosen to reach 40.0 °C. The temperature reading of the circuit was calibrated and adjusted using parameter P4 to match the readings of a reference digital thermometer.

### Force sensing unit

**Fig. 4:**
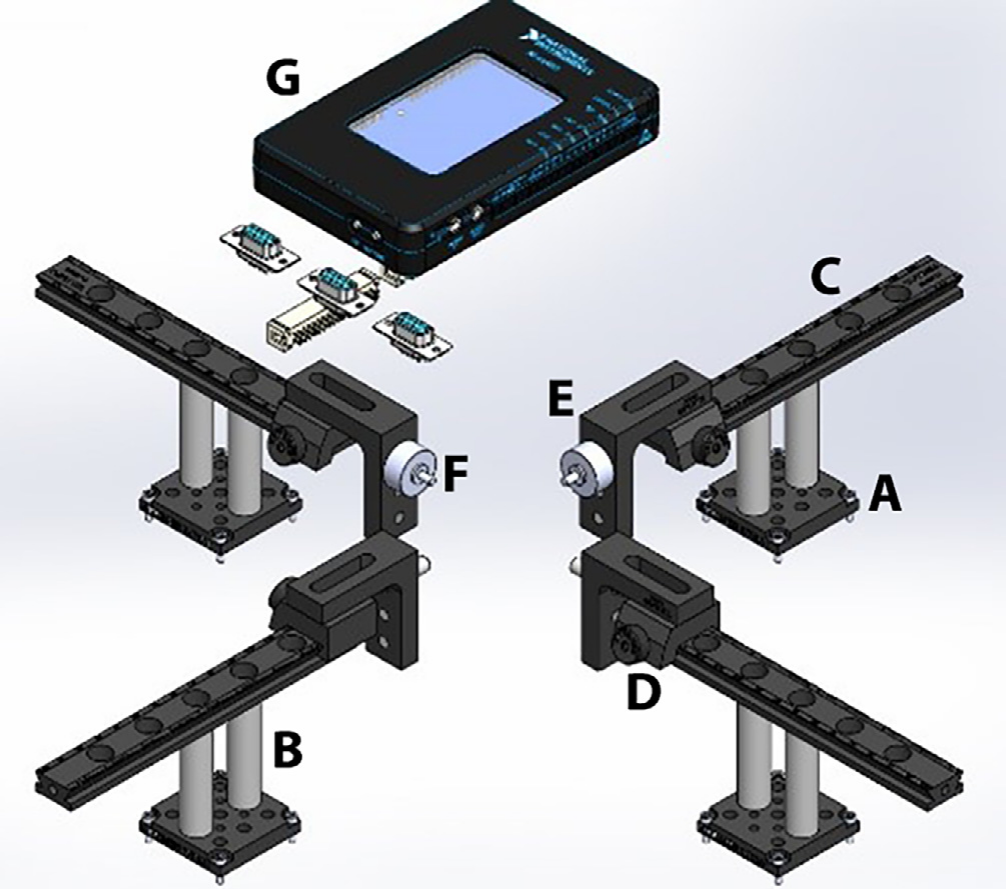
Force sensing unit. (A) Linear actuator adapter plate (MTSA1), (B) stainless steel posts, (C) actuation arm - dovetail optical rail (RLA0600), (D) rail carrier (RC1), (E) right angle bracket (AB90C), (F) sub-miniature load cell (Honeywell, model 31, 2000 g), (G) portable reconfigurable I/O device (myRIO-1900, National instruments).

Each linear actuator has an adapter plate (MTSA1) and two stainless steel posts that connect the device to an actuation arm (Fig. 4). The actuation arm comprises a 6 in. dovetail optical rail (RLA0600), a rail carrier (RC1) and a right angle bracket (AB90C). The carrier can be placed onto the rail anywhere along its length without requiring access to the rail ends. The spring-loaded plunger built into the locking thumbscrew provides wobble-free translation as the carrier slides along the rail. A low-friction joint between the right angle bracket and the rail carrier was implemented using two ball bearings and a stainless steel dowel pin, so that the angle bracket can rotate freely. A tread adapter (MSA25) was then used to secure the fixed end of a sub-miniature load cell (Honeywell, model 31, 2000 g) to one of the ¼”-20 threaded holes in the bracket. The movable end of the load cell was directly connected to the sample clamp. The cable from the load cell was linked to a load cell signal conditioner and digitizing circuit (HX711 Sensor) using a DB-09 connector, so that the load cell can be easily replaced if needed. The HX711 board has a full Wheatstone bridge configuration to sense changes in the load cell, followed by the HX711 microchip, a precision 24-bit analog to digital converter with low-noise programmable gain amplifier. The HX711 uses a two-wire interface (Clock and Data) for synchronization and data transfer (80SPS) with a GPIO. Here we use a portable reconfigurable I/O device (myRIO-1900, National instruments) for real time acquisition of load cell data from the HX711 board, and for transferring the data from the myRIO-1900 into a computer for recording and visualization of data using a graphical user interface in LabView.

### Sample gripping mechanism

**Fig. 5:**
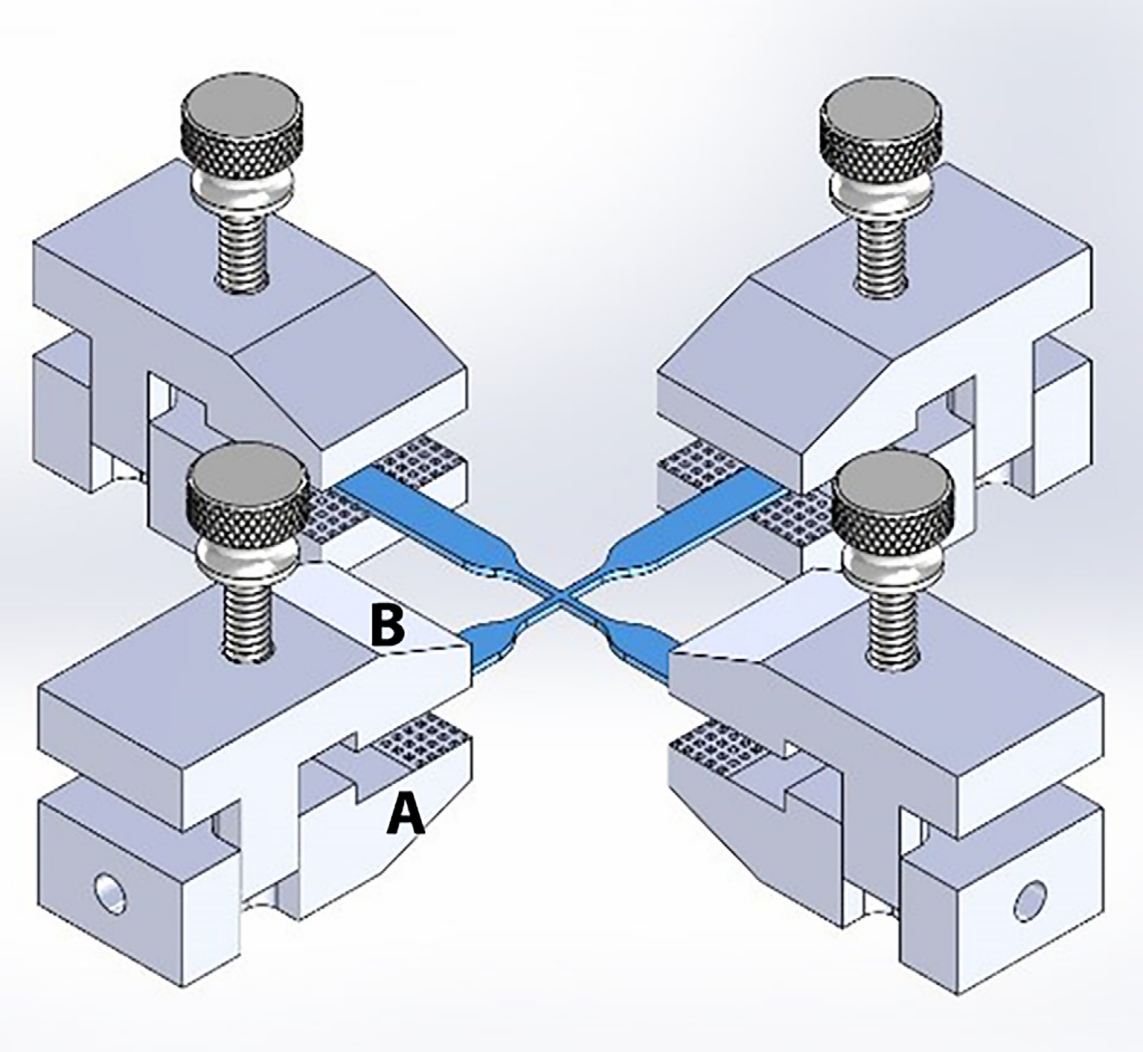
Sample gripping mechanism. (A) aluminum tissue clamp bottom jaw, (B) top jaw, (C) biaxial sample.

The gripping mechanism consists of four small custom made clamps (Fig. 5). Each clamp comprises a bottom jaw, a top closing jaw, and one thumbscrew. The bottom piece is firmly attached to the mobile end of the load cell or the right angle bracket. The top jaw of the clamp can be moved up and down, but remains well aligned to the bottom piece due to a vertical slot and pin built in within the clamp design. The thumbscrew secures the two jaws of the clamp, while maintaining their surfaces well aligned and parallel to provide a uniform holding force on the sample. The top portion of the clamp is removed prior to carefully placing and aligning the sample on top of the bottom jaw. Then, the top jaw is placed and secured via the clamp screw. We manufactured clamps made of acrylic, aluminum and 3D printed using ABS. While all these materials were effective when testing soft materials and tissues, the aluminum clamps are in general considered more durable than the other tested materials.

### Digital image correlation system

**Fig. 6:**
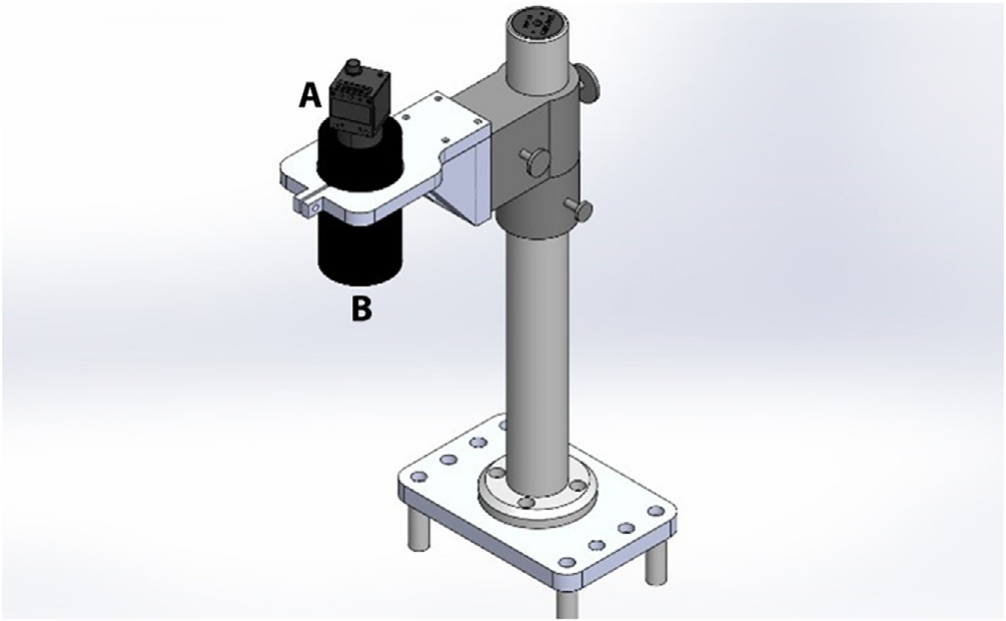
Digital image correlation. (A) high-resolution camera (FLIR Blackfly S, BFS-U3-200S6C-C) and (B) Machine Vision Lens (Tamron M111FM25).

Throughout a uniaxial or biaxial test, images are taken within a Region of Interest (ROI) of the sample, and both deformation and strains are calculated within the ROI using Digital Image Correlation (DIC). This subsystem comprises a high-resolution camera (FLIR Blackfly S, BFS-U3-200S6C-C, 20Mp, 18FPS, CMOS Sony IMX183 sensor) and a Machine Vision Lens (Tamron M111FM25), which are positioned directly above the testing chamber using a custom camera holder (Fig. 6). The camera is connected to the LabVIEW program via a USB3 Vision V1.0 connection port. Within the program, the NI-IMAQ module is utilized to acquire images for the entire duration of the test. Once the test is completed, the images are loaded into the image analyzer program, GOM Correlate (https://www.gom.com/en/). This program is used to calculate and display the strain map experienced by the specimen throughout the duration of the experiment.

### Graphical user Interface

**Fig. 7:**
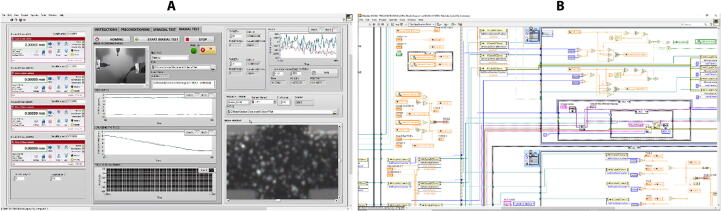
Graphical user interface. (A) LabView front panel used for operation of the system and visualization of data, and (B) LabView block diagram of the operational system.

A graphical user interface (GUI) was developed in LabView to control the operation of the system (Fig. 7). The GUI is split into three main sections, the hardware driver section on the left of the GUI presents the software interface used for communication with the DC servo controller hardware and to set up the working velocity and acceleration of actuators. The middle section of the GUI comprises a Tab control that contains three tabs for either performing preconditioning, uniaxial or biaxial tests. Finally, the right hand side of the GUI has the control boxes where the user can input the desired X and/or Y displacement(s) range, a graph to visualize the measured X and/or Y force(s), and a control to tare the force reading(s) prior to initiate a test. The GUI also has user controls for defining the name and folder where images will be recorded, and a window with real time image of the field of view prior to initiate a test. Data is recorded using the TDMS format (Labview, National Instruments), and images are stored as JPEG. Force, displacement, and time can be easily exported into Matlab for analysis of engineering stresses and strains. The sequence of images taken during the test are also exported into GOM Correlate Software to calculate the true stress and strain in the center region of interest in the sample.

### Key aspects of the system

Overall, the BiMaTS is a low cost device (Table 3) with high precision and accuracy of motion, that offers a large range of displacement necessary for the mechanical testing of samples up to rupture in dry or under immersion conditions with temperature control. The system can perform uniaxial and biaxial tensile testing, is fully automated and customizable by the researcher. The BiMaTS has the capability of characterizing the tensile properties of soft materials in the following manner:

● The system has large displacement range to test samples under uniaxial or biaxial configuration up to rupture
● The small load cell rating allows accurate measurement of reaction forces in soft samples, and it is fully customizable to higher load ranges
● High-resolution images and DIC method are used for non-invasive assessment of the specimen’s strain map
● Fully automated system with configurable parameters (e.g. range of motion, velocity, acceleration, preconditioning cycles, images field of view, etc.)
● The clamps have small weight, are self-aligning, minimize slippage as well as sample rupture at the interface with the grip
● Integrated temperature control system for immersion testing while maintaining physiological conditions
● Cost is under **$15,000** which is significantly lower than commercial biaxial testing systems

## Design files

### Design files summary

**Table 2:** Design files name, type, open source license and repository location.

Design file name	File type	Open source license	Location of the file
Testing platform			
Breadboard	CAD	CC-BY-4.0	https://doi.org/10.17632/8sbwf397jk.1
Back-end Plate	CAD	CC-BY-4.0	https://doi.org/10.17632/8sbwf397jk.1
Base Plate for Stage	CAD	CC-BY-4.0	https://doi.org/10.17632/8sbwf397jk.1
Motorized Translation Stage	CAD	CC-BY-4.0	https://doi.org/10.17632/8sbwf397jk.1
USB Controller Hub and Power Supply for Six K-Cubes	CAD	CC-BY-4.0	https://doi.org/10.17632/8sbwf397jk.1
K-Cube Brushed DC Servo Motor Controller	CAD	CC-BY-4.0	https://doi.org/10.17632/8sbwf397jk.1
Round standoff 0.5 in	CAD	CC-BY-4.0	https://doi.org/10.17632/8sbwf397jk.1
Round standoff 1.25 in (0.75 + 0.5 in)	CAD	CC-BY-4.0	https://doi.org/10.17632/8sbwf397jk.1
Round standoff 1.5 in	CAD	CC-BY-4.0	https://doi.org/10.17632/8sbwf397jk.1
Round standoff 2.0 in	CAD	CC-BY-4.0	https://doi.org/10.17632/8sbwf397jk.1
Front Panel	CAD	CC-BY-4.0	https://doi.org/10.17632/8sbwf397jk.1
Left Panel	CAD	CC-BY-4.0	https://doi.org/10.17632/8sbwf397jk.1
Right Panel	CAD	CC-BY-4.0	https://doi.org/10.17632/8sbwf397jk.1
Back Panel	CAD	CC-BY-4.0	https://doi.org/10.17632/8sbwf397jk.1
Back Panel Top	CAD	CC-BY-4.0	https://doi.org/10.17632/8sbwf397jk.1
Case cover middle	CAD	CC-BY-4.0	https://doi.org/10.17632/8sbwf397jk.1
Case cover Top	CAD	CC-BY-4.0	https://doi.org/10.17632/8sbwf397jk.1
Corner adapter	CAD	CC-BY-4.0	https://doi.org/10.17632/8sbwf397jk.1
Cap Nuts	CAD	CC-BY-4.0	https://doi.org/10.17632/8sbwf397jk.1
Power Module	CAD	CC-BY-4.0	https://doi.org/10.17632/8sbwf397jk.1
Flanged Screw-to-Expand Inserts for Plastic	CAD	CC-BY-4.0	https://doi.org/10.17632/8sbwf397jk.1
Flanged Button Head Screws	CAD	CC-BY-4.0	https://doi.org/10.17632/8sbwf397jk.1
Specimen Testing Chamber			
Testing chamber	CAD	CC-BY-4.0	https://doi.org/10.17632/8sbwf397jk.1
Pedestal Pillar Post 1.5in	CAD	CC-BY-4.0	https://doi.org/10.17632/8sbwf397jk.1
Stainless steel plate	CAD	CC-BY-4.0	https://doi.org/10.17632/8sbwf397jk.1
Peltier plate	CAD	CC-BY-4.0	https://doi.org/10.17632/8sbwf397jk.1
Thermostat	CAD	CC-BY-4.0	https://doi.org/10.17632/8sbwf397jk.1
Thermostat Bracket	CAD	CC-BY-4.0	https://doi.org/10.17632/8sbwf397jk.1
Temperature sensor	CAD	CC-BY-4.0	https://doi.org/10.17632/8sbwf397jk.1
tmp60 power supply	CAD	CC-BY-4.0	https://doi.org/10.17632/8sbwf397jk.1
tmp30 power supply	CAD	CC-BY-4.0	https://doi.org/10.17632/8sbwf397jk.1
Force sensing unit			
Adapter Plate for MTS50 Stages	CAD	CC-BY-4.0	https://doi.org/10.17632/8sbwf397jk.1
Dovetail Optical Rail	CAD	CC-BY-4.0	https://doi.org/10.17632/8sbwf397jk.1
Dovetail Rail Carrier	CAD	CC-BY-4.0	https://doi.org/10.17632/8sbwf397jk.1
Right-Angle Bracket	CAD	CC-BY-4.0	https://doi.org/10.17632/8sbwf397jk.1
Thread Adapter	CAD	CC-BY-4.0	https://doi.org/10.17632/8sbwf397jk.1
Honeywell load sensor	CAD	CC-BY-4.0	https://doi.org/10.17632/8sbwf397jk.1
National Instruments -myRIO-1900	CAD	CC-BY-4.0	https://doi.org/10.17632/8sbwf397jk.1
HX711 Weighing Sensor	CAD	CC-BY-4.0	https://doi.org/10.17632/8sbwf397jk.1
DB-9F-2	CAD	CC-BY-4.0	https://doi.org/10.17632/8sbwf397jk.1
CONN HEADER 34POS IDC	CAD	CC-BY-4.0	https://doi.org/10.17632/8sbwf397jk.1
Sample gripping mechanism			
Metal Grip Top	CAD	CC-BY-4.0	https://doi.org/10.17632/8sbwf397jk.1
Metal Grip Bottom	CAD	CC-BY-4.0	https://doi.org/10.17632/8sbwf397jk.1
Thumbscrew	CAD	CC-BY-4.0	https://doi.org/10.17632/8sbwf397jk.1
Type-D biaxial sample	CAD	CC-BY-4.0	https://doi.org/10.17632/8sbwf397jk.1
Sample_Holder_Base	CAD	CC-BY-4.0	https://doi.org/10.17632/8sbwf397jk.1
Sample_Holder_Top	CAD	CC-BY-4.0	https://doi.org/10.17632/8sbwf397jk.1
Optical Strain mapping			
Camera Base Plate	CAD	CC-BY-4.0	https://doi.org/10.17632/8sbwf397jk.1
Dynamically Damped Post	CAD	CC-BY-4.0	https://doi.org/10.17632/8sbwf397jk.1
Lens Holder Assembly	CAD	CC-BY-4.0	https://doi.org/10.17632/8sbwf397jk.1
Camera	CAD	CC-BY-4.0	https://doi.org/10.17632/8sbwf397jk.1
Lens	CAD	CC-BY-4.0	https://doi.org/10.17632/8sbwf397jk.1

## Bill of materials

**Table 3:** Bill of materials, designator, component, unit cost, total cost, source and type of material.

Designator	Component	Number	Cost per unit -currency	Total cost - currency	Source of materials	Material type
Testing Platform						
Breadboard	MB18 18″ x 18″ x 1/2″, 1/4″-20 Taps, thorlabs	1	$281.56	$281.56	https://www.thorlabs.com/thorproduct.cfm?partnumber=MB18	Metal
Back-end Plate	8560 K266	1	$59.08	$59.08	https://www.mcmaster.com/catalog/127/3896	Acrylic
Base Plate for Stage	MTS50A-Z8	4	$86.03	$344.12	https://www.thorlabs.com/newgrouppage9.cfm?objectgroup_id=3002&pn=MTS50A-Z8#3116	Metal
Motorized Translation Stage	MTS50-Z8 50 mm (1.97″), 8–32 and 4–40 Taps	4	$1,151.38	$4,605.52	https://www.thorlabs.com/newgrouppage9.cfm?objectgroup_id=3002&pn=MTS50-Z8#3006	Metal
USB Controller Hub and Power Supply for Six K-Cubes	KCH601	1	$635.20	$635.20	https://www.thorlabs.com/newgrouppage9.cfm?objectgroup_id=2424&pn=KCH601#13030	Electronic Comp
K-Cube Brushed DC Servo Motor Controller	KDC101	4	$677.41	$2,709.64	https://www.thorlabs.com/newgrouppage9.cfm?objectgroup_id=2419&pn=KDC101#5077	Electronic Comp
Standoff 0.5 in	91125A382	28	$3.20	$89.60	https://www.mcmaster.com/catalog/127/3539/	Metal
Standoff 0.75 in	91125A392	24	$3.41	$81.84	https://www.mcmaster.com/catalog/127/3539/	Metal
Standoff 1.5 in	91125A422	31	$3.92	$121.52	https://www.mcmaster.com/catalog/127/3539/	Metal
Standoff 2.0 in	91125A652	12	$3.95	$47.40	https://www.mcmaster.com/catalog/127/3539/	
Front Panel	1227 T259	2ft	$3.48	$6.96	https://www.mcmaster.com/catalog/127/3897/	Acrylic
Left Panel	1227 T259	2ft	$3.48	$6.96	https://www.mcmaster.com/catalog/127/3897/	Acrylic
Right Panel	1227 T259	2ft	$3.48	$6.96	https://www.mcmaster.com/catalog/127/3897/	Acrylic
Back Panel	1227 T259	2ft	$3.48	$6.96	https://www.mcmaster.com/catalog/127/3897/	Acrylic
Back Panel Top	1227 T259	2ft	$3.48	$6.96	https://www.mcmaster.com/catalog/127/3897/	Acrylic
Case cover middle	8589 K83	1	$35.42	$35.42	https://www.mcmaster.com/catalog/127/3899/	Acrylic
Case cover Top	8589 K83	1	$35.42	$35.42	https://www.mcmaster.com/catalog/127/3899/	Acrylic
Corner adapter	1227 T529	2ft	$6.67	$13.34	https://www.mcmaster.com/catalog/127/3897	Acrylic
Cap Nuts	91855A520	2 pk	$8.17	$16.34	https://www.mcmaster.com/catalog/127/3460/	Metal
Power Module	DD11.0111.1111	1	$11.86	$11.86	https://www.newark.com/schurter/dd11-0111-1111/c14-inlet-250vac-10a-quick-connect/dp/48M7010	Electric comp
Flanged Screw-to-Expand Inserts for Plastic	95110A113	2 pk	$11.67	$23.34	https://www.mcmaster.com/catalog/127/3568/	Metal
Flanged Button Head Screws	96660A156	2 pk	$11.69	$23.38	https://www.mcmaster.com/catalog/127/3268/	Metal
Sample testing chamber						
Testing Chamber	8619 K491	1	$30.77	$30.77	https://www.mcmaster.com/catalog/127/3924/	Polyethylene HDPE
Pedestal Pillar Post 1.5in	TRP1.5	4	$21.64	$86.56	https://www.thorlabs.com/newgrouppage9.cfm?objectgroup_id=10491&pn=TRP1.5#10495	Metal
Stainless Steel plate, 0.024″ thick 6″x6″	8983 K111	1	$3.87	$3.87	https://www.mcmaster.com/8983K111/	Metal
Peltier plate	TEC1-12706	1	$9.99	$9.99	https://www.amazon.com/DAOKI-TEC1-12706-Heatsink-Thermoelectric-Cooling/dp/B00XT0OZY0/ref=sr_1_23?dchild=1&keywords=peltier+plate&qid=1631740703&s=industrial&sr=1-23	component
Thermostat and temperature sensor	W1209	1	$7.89	$7.89	https://www.amazon.com/Temperature-Controller-Thermostat-Envistia-Mall/dp/B07N3Y2M3Z/ref=sr_1_14?dchild=1&keywords=W1209&qid=1631740609&s=industrial&sr=1-14	Electronic component
Thermostat Bracket	9115 K43	1	$36.39	$36.39	https://www.mcmaster.com/acrylic/thickness~1-2/width~1/length~12/	Acrylic
tmp60 power supply	TMP 60,112	1	$71.90	$71.90	https://www.digikey.com/en/products/detail/traco-power/TMP-60112/9343878	Electric comp
tmp30 power supply	TMP 30,112	1	$55.30	$55.30	https://www.digikey.com/en/products/detail/traco-power/TMP-30112/9343860	Electric comp
Force sensing unit						
Adapter Plate for Stage	MTSA1	4	$47.35	$189.40	https://www.thorlabs.com/newgrouppage9.cfm?objectgroup_id=3423&pn=MTSA1#7391	Metal
Dovetail Optical Rail	RLA0600	4	$45.72	$182.88	https://www.thorlabs.com/newgrouppage9.cfm?objectgroup_id=30&pn=RLA0600#8294	Metal
Dovetail Rail Carrier	RC1	4	$26.94	$107.76	https://www.thorlabs.com/newgrouppage9.cfm?objectgroup_id=8295&pn=RC1#8296	Metal
Slim Right-Angle Bracket	AB90C	4	$27.85	$111.40	https://www.thorlabs.com/newgrouppage9.cfm?objectgroup_id=223&pn=AB90C#2119	Metal
National Instruments -myRIO-1900	782692–01	1	$571.50	$571.50	https://www.ni.com/en-us/shop/hardware/products/myrio-student-embedded-device.html?modelId=125751	Electronic component
Honeywell load sensor	060–1432-07	2	$1,288.77	$2,577.54	https://www.digikey.com/en/products/detail/honeywell-sensing-and-productivity-solutions-t-m/060–1432-07/5055799	Sensor
Thread Adapter	AE6E25E	4	$4.53	$18.12	https://www.thorlabs.com/newgrouppage9.cfm?objectgroup_id=1745&pn=AE6E25E#1439	Metal
HX711 Weighing Sensor	HX711	3	$5.69 / 3 pc	$5.69	https://www.amazon.com/HiLetgo-Weighing-Dual-Channel-Precision-Pressure/dp/B00XRRNCOO/ref=pd_lpo_2?pd_rd_i=B00XRRNCOO&psc=1	Electronic component
DB-9F-2	2301838–1	3	$2.21	$6.63	https://www.digikey.com/en/products/detail/te-connectivity-amp-connectors/2301838-1/7776535	Connector
CONN HEADER 34POS IDC	732–5457-ND	2	$5.86	$11.72	https://www.digikey.com/en/products/detail/w%C3%BCrth-elektronik/61203425821/4846942	Connector
34 Position Cable Assembly 0.500′	H3CCH-3406G	2	$1.57	$3.14	https://www.digikey.com/en/products/detail/assmann-wsw-components/H3CCH-3406G/1218569	Cable assembly
Sample gripping mech.						
Metal Grip Top	8975 K618	1	$4.49	$4.49	https://www.mcmaster.com/catalog/127/3979/	Metal
Metal Grip Bottom	8975 K618	1	$4.49	$4.49	https://www.mcmaster.com/catalog/127/3979/	Metal
Type-D biaxial sample	Sylgard 184	1/10	$15.30	$15.30	https://www.amazon.com/Electron-Microscopy-Sciences-Sylgard-184/dp/B00K335I0G	PDMS
Sample Holder Base	1227 T459	1	$6.07	$6.07	https://www.mcmaster.com/1227T459/	Acrylic
Sample Holder Top	8531 K21	1	$18.96	$18.96	https://www.mcmaster.com/8531K21/	Acrylic
TOptical strain mapping						
Dynamically Damped Post	DP14A-POST	1	$219.67	$219.67	https://www.thorlabs.com/newgrouppage9.cfm?objectgroup_id=170&pn=DP14A#170	Metal
Lens Holder Assembly	8560 K265	1	$33.98	$33.98	https://www.mcmaster.com/acrylic/thickness~1-2/	Acrylic
Lens	Tamron M111FM25	1	$579.00	$579.00	https://www.bhphotovideo.com/c/product/1181229-REG/tamron_m111fm50_12mp_50mm_fixed_focal.html	Component
Camera	BFS-U3-200S6C-C	1	$729.00	$729.00	https://www.flir.com/products/blackfly-s-usb3/?model=BFS-U3-200S6C-C	Electronic component

## Build Instructions

### Testing platform

**Fig. 8:**
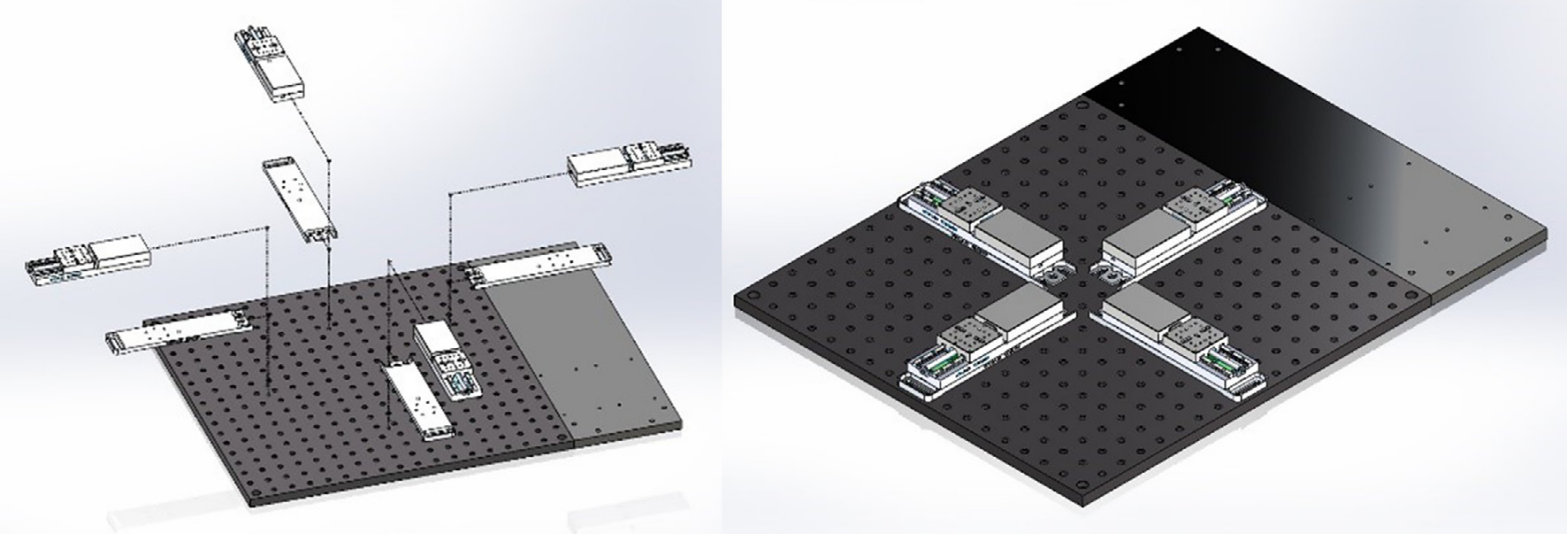
Testing platform base. The perforated optical breadboard and back-end plate create a 24″ x 24″ footprint for building the system. The Breadboard Mounting Adapters (MTS50A-Z8) are positioned in a cruciform shape on the center of the breadboard, and the linear actuators (MTS50-Z8) are attached to mounting adapters.

The testing platform (Fig. 8) was built by selecting off-the-shelf parts and components that can easily be assembled and reconfigured if needed. The 18x18″ perforated optical breadboard ensures adequate alignment of the four linear actuators in a cruciform configuration. A 18x6″ plate was added on the back-end of the system to increase the surface area to 18″x24″ and provide enough space for the Servo Motor Drivers. The linear actuators (MTS50-Z8) are attached to the breadboard using Breadboard Mounting Adapters (MTS50A-Z8).

**Fig. 9:**
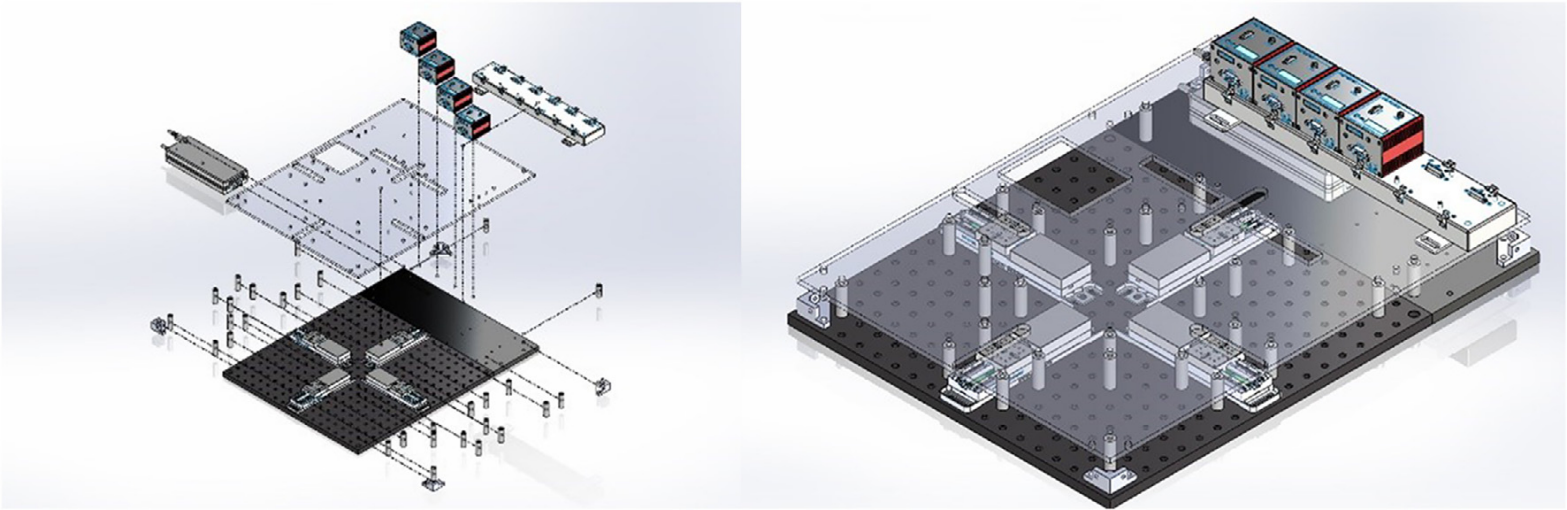
Testing platform plate. The acrylic plate was attached to the system using ¼-20 female threaded round standoffs and ¼-20 threaded rods. The USB Controller Hub is placed on top of the acrylic plate at the back-end of the system. The USB Controller Hub power supply is found under the controller hub.

The linear actuators are physically isolated from other components by using an acrylic plate supported by several round standoffs (Fig. 9). This separation plate also holds in place the USB Controller Hub where the four DC Servo Motor Drivers are connected. Below the USB Controller Hub the power supply can be found. The system was designed in SolidWorks, and flat components, such as the acrylic plate, were manufactured using a CO_2_ laser cutter. Other non-flat components, such as the acrylic corner brackets were in turn fabricated using a Modela Pro II MDX-540 3D milling machine. All components were attached to the breadboard using ¼-20 female threaded round standoffs and ¼-20 threaded rods.

### Specimen testing chamber

**Fig. 10:**
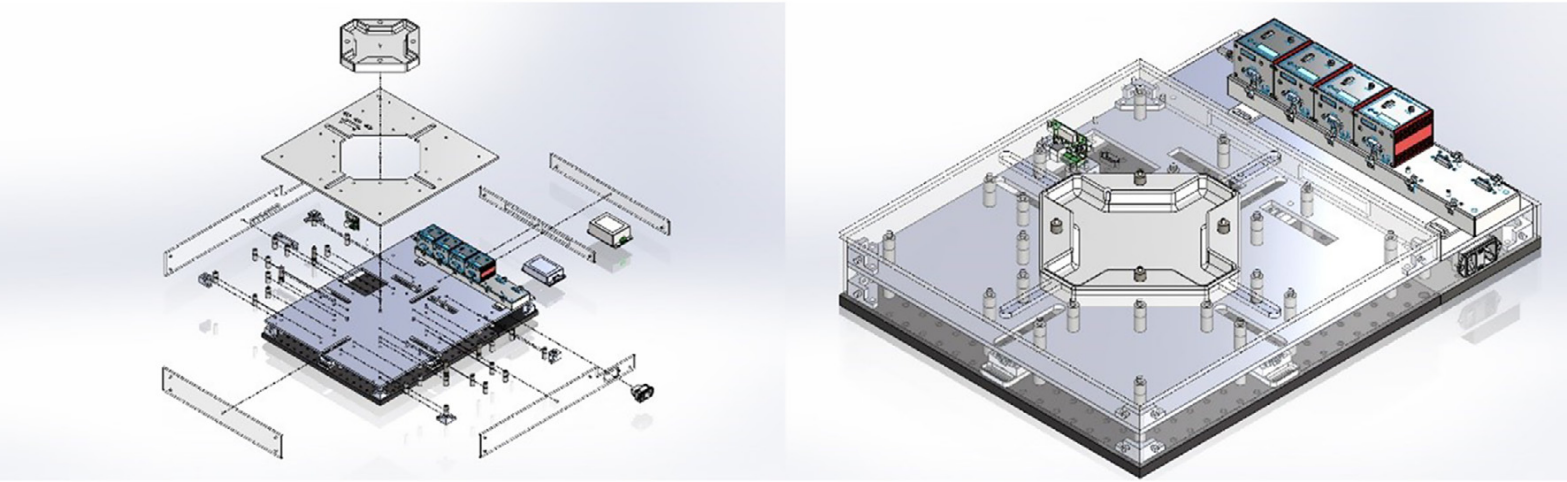
Specimen testing chamber building. The PTFE testing chamber and top acrylic plate are hold in place by stainless steel threaded standoffs and threaded rods. The temperature board is attached to the top plate using a rectangular bracket fabricated in the milling machine. The two power supplies used for the Peltier plate and the W1209 control board are secured on the back-end plate under the USB hub. The temperature sensor is pressed fit within a sensor hole carved within the testing chamber.

The specimen testing chamber was manufactured using a MDX-540 milling machine. The SolidPart file from SolidWorks was transformed into STL format and loaded into SRP player to create the milling paths. The testing chamber was cut from a 12″x12″x2″ Polytetrafluoroethylene (PTFE) stock. The top and lateral side acrylic plates were also manufactured using the laser cutter. These acrylic plates have all necessary cut through holes to allow the passage of cables, temperature controller, main power switch, movable parts and the testing chamber (Fig. 10). The middle and top plates between the linear actuators and the testing chamber helps isolate the motors from the heating actuator and the fluid in the testing chamber. The two plates are hold in place by stainless steel threaded studs. The temperature board is attached to the top plate using a rectangular bracket fabricated in the milling machine. The two power supplies used for the Peltier plate and the W1209 control board are secured on the extension plate under the USB hub. The heating side of the Peltier thermal actuator is attached using thermal paste to a 4 × 4 × 0.024 in stainless steel plate. The temperature sensor is pressed fit within a sensor hole carved within the testing chamber.

### Force sensing unit

**Fig. 11:**
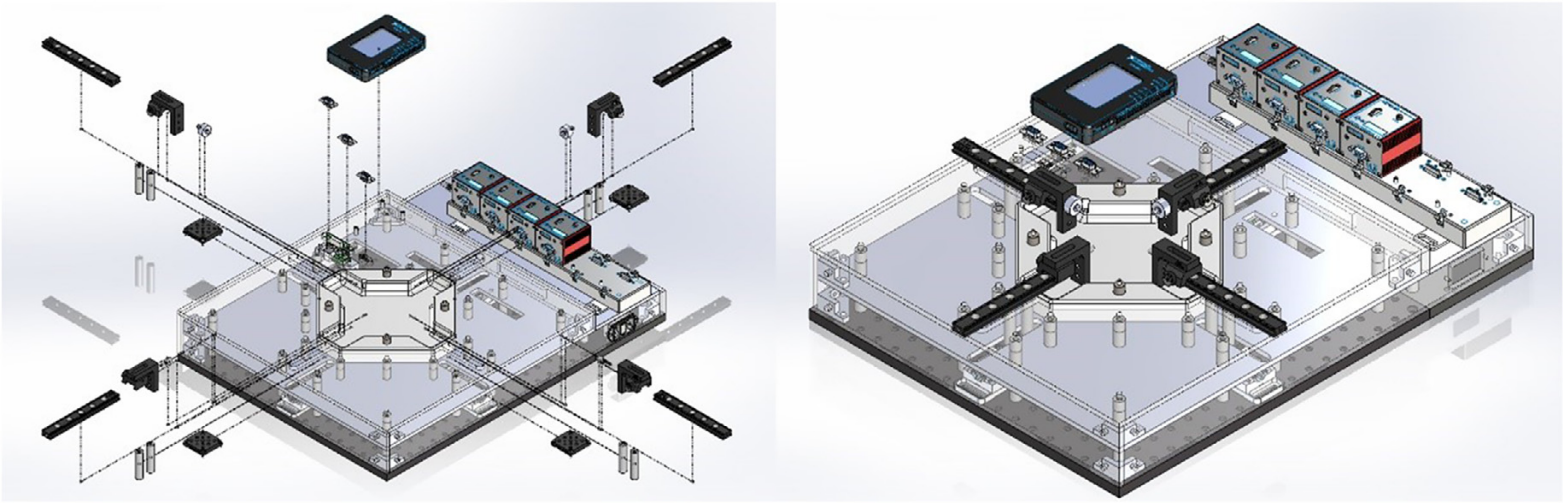
Force Sensing Unit. An MTSA1 adapter plate is attached to the movable part of each linear actuator and supports two round standoffs that link the actuators to the dovetail optical rail (RLA0600). The adjustable rail carrier has a low friction hinge connecting to the right angle bracket (AB90C). A MSA25 tread adapter was inserted into the right angle bracket to attach a sub-miniature load cell (Honeywell, model 31). The load cell is connected to a signal conditioner and digitizing circuit (HX711 Sensor). The digital outputs of the HX711 board are connected to a portable reconfigurable I/O device (myRIO-1900 board).

An MTSA1 adapter plate was attached to the movable part of each linear actuator, and two stainless steel, female threaded round standoffs, are in turn connected to the MTSA1 adapter using ¼”-20 threaded rods (Fig. 11). The dovetail optical rail (RLA0600) is secured on top of the standoffs using ¼”-20 stainless steel socket head screws. A hinge system was built between the rail carrier (RC1) and a right angle bracket (AB90C) using two ball bearings (Trade Number R3, for 3/16″ shaft diameter) and a stainless steel dowel pin (3/16″ diameter, 7/16″ long). To create this hinge, a ½” diameter, 3/16″ long cut was made with the milling machine on the bottom face of the dovetail optical rail and on the counterbore mounting slot of the right angle bracket. Then a ball bearing was press fit attached into each cut, and the two movable pieces were connected together placing the dowel pin through the center opening of the ball bearings. The dovetail optical rail was placed onto the rail, while keeping the right angle bracket inside the testing chamber. The right angle bracket has three ¼”-20 tapped holes, and the MSA25 tread adapter was inserted into one of them to attach the fixed end of a sub-miniature load cell (Honeywell, model 31, 2000 g). The cable from the load cell was soldered to a DB-09 male connector so that the load cell can be easily replaced if needed, and a matching DB-09 female connector was installed on the top acrylic plate of the system. The DB-09 female terminals are connected inside the system to a load cell signal conditioner and digitizing circuit (HX711 Sensor). Three DB-09 female connectors are attached to the top acrylic plate and wired to the inputs of three HX711 load sensing boards. Each HX711 board has a full Wheatstone bridge configuration to sense changes in the load cell, followed by the HX711 microchip, a precision 24-bit analog to digital converter with low-noise programmable gain amplifiers. The HX711 uses a two-wire interface (Clock and Data) for synchronization and data transfer (80SPS) with a GPIO. The digital outputs of the HX711 board are connected to ribbon cable connectors located on the left side wall of the system. A Ribbon cable then links the digital outputs of the system to the myRIO-1900 board. This portable reconfigurable I/O device allows for real time acquisition of load cell data from the HX711 board into a computer for recording and visualization of data. The myRIO-1900 board is attached to the cover plate of the system next to the DB-09 female connectors.

### Tissue gripping mechanism

**Fig. 12:**
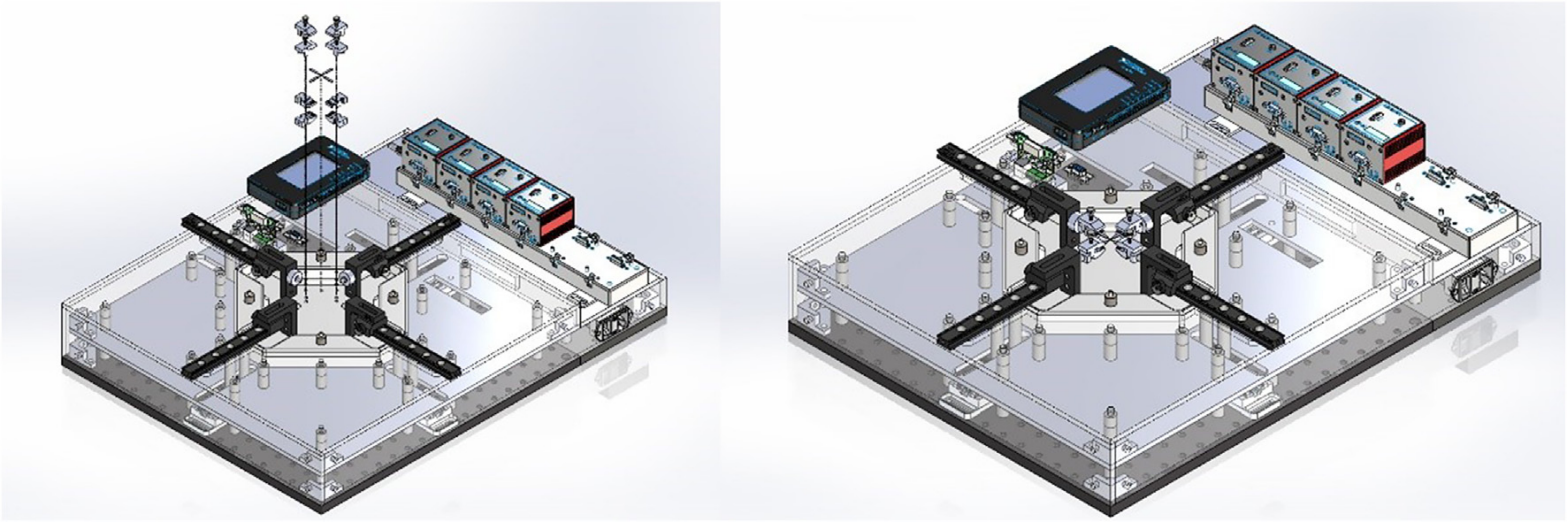
Tissue gripping mechanism. Small custom-made clamps have two opposing jaws with small pyramids to reduce the possibility of sample slippage. The jaws of the clamp design are self-aligned by the vertical slot and pin built in within the clamp design. A uniform holding force is achieved using a thumbscrew that secures the two jaws of the clamp.

The small custom made clamps were first prototyped in ABS plastic using a 3D printer, followed by manufacturing using the 3D milling machine using acrylic and aluminum. Both clamp jaws have small pyramids to increase the friction between the clamp and the sample, and thus reduce the possibility of slippage. Different designs were tested until obtaining a clamp with the smallest footprint and volume, so to minimize the weight that is attached to the end of the load cell. The jaws of the final clamp design remain always well aligned by the vertical slot and pin built in within the clamp design. The thumbscrew secures the two jaws of the clamp, while maintaining their surfaces well aligned and parallel to provide a uniform holding force on the sample (Fig. 12).

**Fig. 13:**
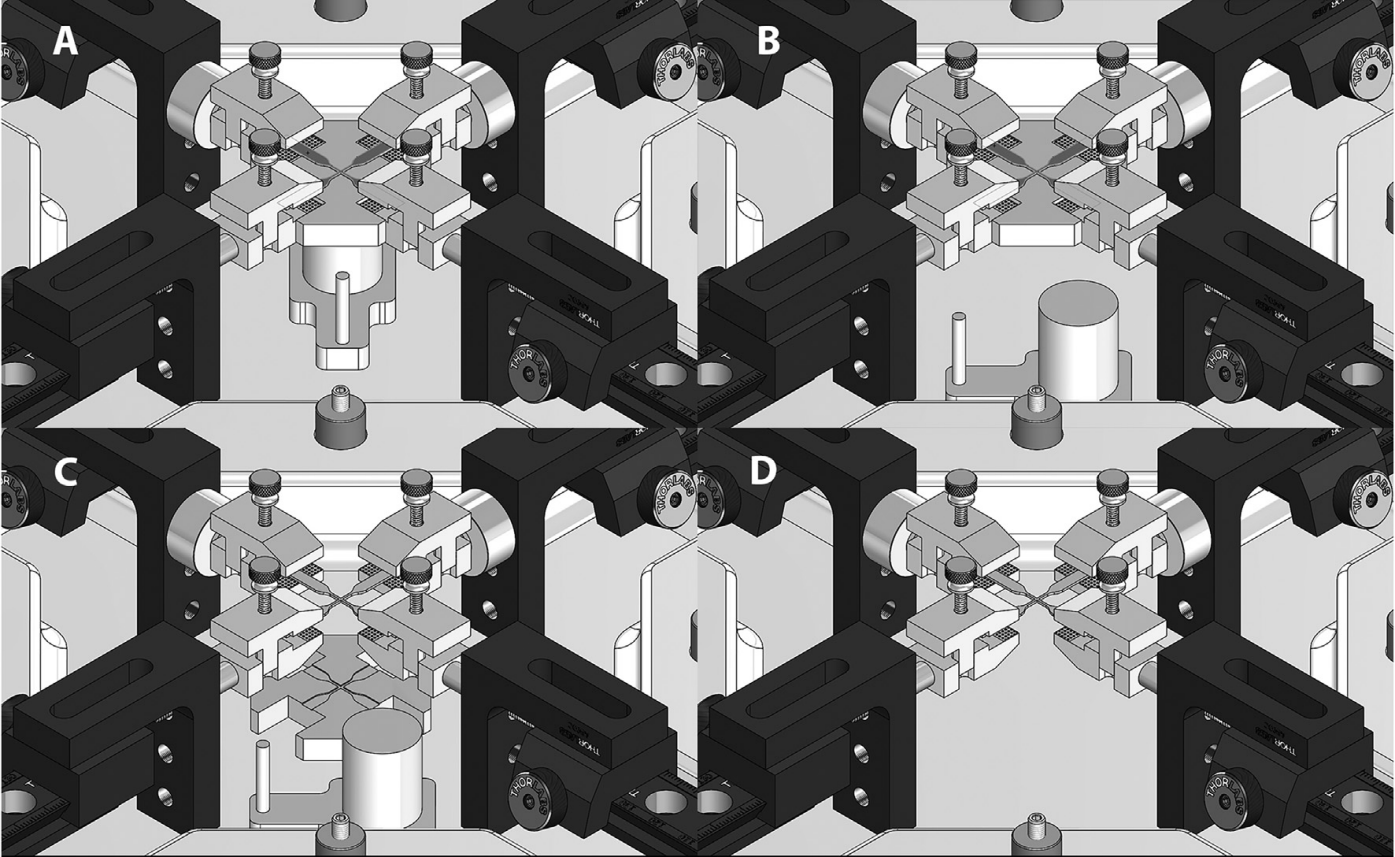
Tissue holding and alignment system. The sample holder has a top holder and a removable bottom holder. (A) The bottom holder component is placed at the center of the testing chamber, the top holder component is placed on top of its bottom counterpart and aligned with the lower part of the clamp. The sample is then placed at the center of the sample holder and the top portion of the clamps are set in place and secured with the thumbscrews. (B) Once the sample is secured, the bottom holder component is removed by sliding it carefully away from the center of the system, and (C) the top holder portion is moved down and (D) removed from the testing chamber. The use of this sample holder allow us to set the sample at the center of the system, well aligned with the actuation axes.

A sample holder was designed to help mounting the sample into the system at the center of the 2 axes (Fig. 13). The sample holder comprises two components, the top holder has a groove with the shape of the biaxial sample, and can hold either uniaxial or biaxial samples. The top holder also has four extruded cuts to hold the bottom part of the clamp in place, aligned with its dovetail optical rail. The bottom holder component has a cylinder, a plate and a handle. Initially, the bottom holder is placed at the center of the testing chamber, the top holder component is placed on top of its bottom counterpart and aligned with the lower part of the clamp. The sample is then placed at the center of the sample holder and the top portion of the clamps are set in place and secured with the thumbscrews. Once the sample is secured, the bottom holder component is removed by sliding it carefully away from the center of the system, and the top holder portion is moved down and removed from the testing chamber. The use of this sample holder allow us to set the sample at the center of the system, well aligned with the actuation axes.

### Digital image correlation system

**Fig. 14:**
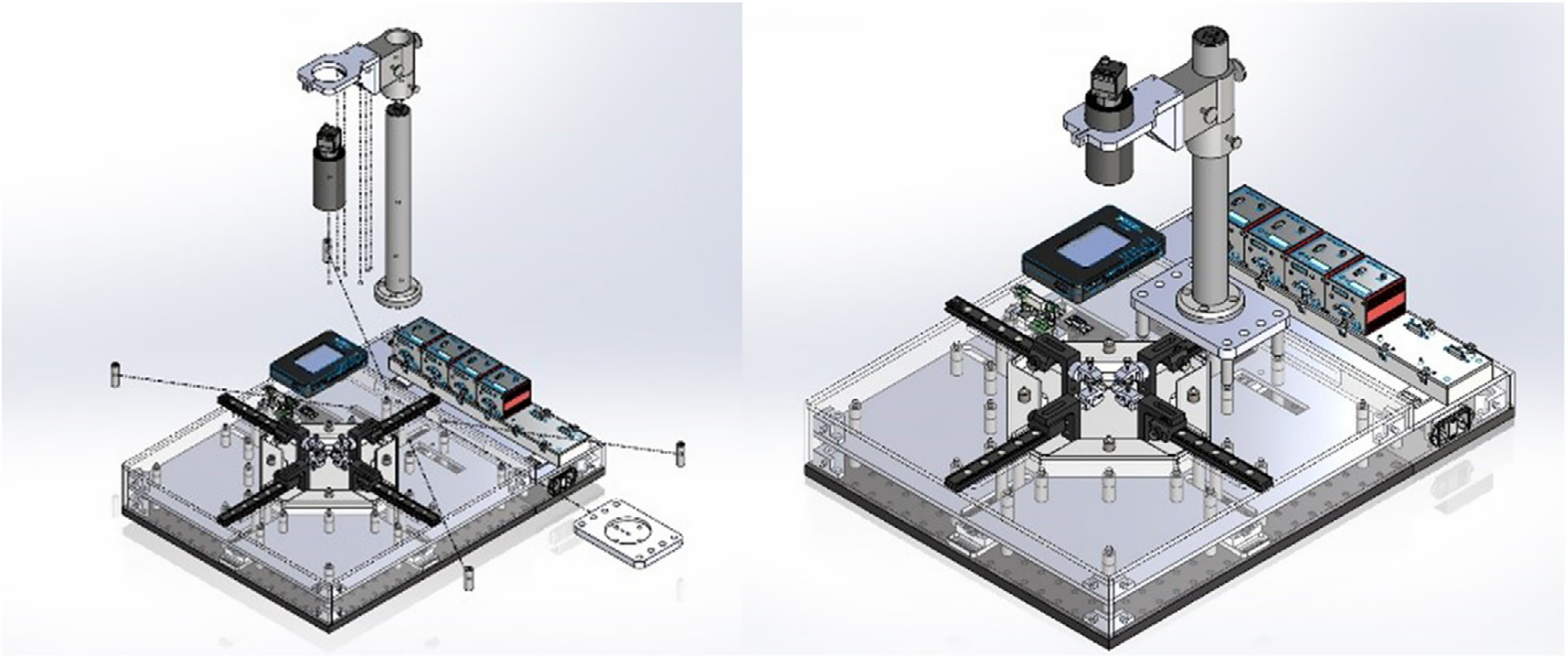
Digital Image correlation system. Four standoff pints support a custom acrylic camera holder plate, a round post, camera holder plate and a lens bracket. The Machine Vision lens is inserted in the camera holder, the digital camera is attached to the back of the lens using a C-mount adapter and connected to the computer using a USB3 with power cable. The ROI in the image and focal plane of the lens can be adjusted by either modifying the position of the camera holder or by adjusting the lens focal distance.

Four standoff pints were attached on top of the top acrylic plate to support a custom acrylic camera holder plate. A round post is secured with four screws into the camera holder plate and a lens bracket was attached to the camera post (Fig. 14). The custom lens holder was manufactured in the 3D milling machine and has an opening through which the Machine Vision lens is inserted and secured using a set screw. The digital camera is attached to the back of the lens using a C-mount adapter and connected to the computer using a USB3 with power cable. The dimensions of the lens holder were chosen so that the center of the camera field of view coincides with the center of the system where the sample will be stretched. The positioning of the holder can be adjusted vertically to modify the ROI in the image, or by modifying the focal plane of the lens.

## Operation Instructions*.*

### Initialization of the system

Turning on the main switch of the system provides power to the USB hub, DC Servo controllers, and the NI MyRIO system. The USB hub and DC servo controllers run an initialization routine to check the communication with the PC, and then stay in standby. The high resolution camera is turned on once its USB cable is connected to the PC. There is no on/off switch for the camera, thus, the USB cable needs to be unplugged to turn off the camera. The communication between these devices and the PC can be set and/or throubleshooted using NIMax software in case of any communication problem. The next step is to run the LabView software interface, and start the Labview project “BiMaTS.lvproj”. This project comprises a virtual interface file that resides in the local PC, and a link to a “MyRIO-1900” target device. In turn, the MyRIO-1900 device has a real time virtual interface file to acquire data from the load cells, and a VI recorded within the FPGA of the target device. It is only required to run the VI in the local PC “BiMATS.vi” to initialize and get started the VIs in the PC, target device and FPGA. The Graphic User Interface will appear on screen, and it is necessary to click on “Run” or select the menu “Operate”, followed by “Run”, or use the hotkeys “CTR + R”. The DC Servo driver will follow a homing sequence and the load cell(s) will start acquiring data that is displayed in the force window. If the test is performed under immersion, the heating system can be switched on to warm up the fluid in the chamber for about 30 min, until the desired temperature is achieved. This warm-up period will also help the load cells to reach a stable operating temperature.

### Mounting samples

Silicone samples are prepared using a mold and curing the material in a laboratory oven. This approach provide samples with consistent shape and size, according to ASTM Standard D412 “Tension Testing for Rubber and Elastomers”. The sample surface is sprayed with acrylic ink droplets for speckle recognition using DIC for strain measurements. DIC is used to obtain more accurate data as it enables measuring the strain directly at the sample’s surface. Depending on the size of the samples being tested, the distance between grips can be adjusted to maintain the region of interest at the center of the camera field of view. The distance between grips is easily adjusted using the dovetail rail carriers, without moving the linear actuator from the zero position. The top section of the clamp is removed and the sample is placed on top of the bottom clamp jaw, and fastened by the top clamp. The small pyramids on the clamp jaw help providing increased friction with the sample to avoid the possibility of slippage. When setting the samples into the grips, ensure to not apply excessive force at the grip-sample interface when manually tightening the screw, to avoid damaging the sample. When working with biaxial samples, fix the sample arms into the grips opposite to each other first, then adjacent grips. The camera lighting can be adjusted at this stage to obtain the best images possible at the center of the sample’s region of interest.

### Operation of software interface for mechanical test

When the LabView project file is started, the project file initializes the software, creates the variables, runs the data acquisition code inside the myRIO interface, the initialization of the camera and the connection between the software and each of the servo driver hardware by reading and comparing the serial number defined for each axis and the serial number embedded in the hardware. The next automated step is to run an initialization routine where the actuators read the desired velocity and acceleration entered by the user, and move the actuator a few millimeters forward, followed by a home cycle, where all the actuators are moved backwards to the zero position. The camera field of view is displayed in the Image Preview window, but they are not recorded in the computer, so that the user can adjust the field of view or the focus of the lens. The system is ready for operation, and once the user has placed the sample in the system, it is recommended to perform a preconditioning of the sample, where the sample is stretched cyclically within a displacement range that would not lead to rupture (e.g. n = 10 cycles, 50% stretch), and will reduce hysteresis and the possibility of sample slippage. The user can select the number of cycles, file name and file path for data and images. The preconditioning can be performed for uniaxial or biaxial tests. After preconditioning of the sample, the user may chose performing a uniaxial or a biaxial test by selecting the corresponding tab in the GUI, next to the right of the preconditioning tab. The user can select the file name and path for recording the force, displacement, time and images being acquired during the test. The user may also choose to run a homing cycle, and return the actuators to the zero position, at the end of a test. Preconditioning, uniaxial and biaxial tabs have an Image Recording window that shows the images that are being stored in the PC hard drive during the test. Once preconditioning is completed, the user will enter the target displacement for the horizontal and/or the vertical direction. The presence of two distinct tabs for the target displacements in the horizontal and vertical directions allows to perform two types of biaxial loading: (1) the target displacement, velocity and acceleration are imposed in the two tensile directions; (2) the target displacement, velocity and acceleration are imposed in one tensile direction while the position of the sample in the other direction is maintained constant. The user has the possibility of easily defining any other desired motion sequence. Tare the load cells (set to zero) before starting the test, to ensure zero offset of the force readings. Force and displacement are acquired simultaneously at a high rate using Real-time VIs at the MyRIO-1900 system, temporally stored in the MyRIO-1900, and then transferred to the PC at a lower rate. Images acquisition and recording were automatically started by the initiation of the test, and automatically stopped when the target range of motion is achieved. Data is recorded using the TDMS format (Labview, National Instruments), and images are stored as JPEG. Force, displacement, and time can be easily exported into Matlab for analysis, and the sequence of images taken during the test are also exported into GOM Correlate Software to calculate engineering stress and strain, and then determine true stress and strain. These experimental data can be used in general for determining the material properties of tissues and soft materials. The non-linear behavior of soft materials can also be used to curve fit constitutive models and characterize their parameters. In particular, the ultimate stress, strength and strain at failure can be determined.

## Validation and performance characterization

### Actuation System: Displacement and velocity

The linear motion performance of the system was characterized using a series of tests. First, to validate each linear stage motion accuracy, we compared different target displacement values introduced in the LabView interface versus measurements of displacement recorded using a digital caliper. We performed five tests with different target displacements in the x- and y- directions, and measured the initial separation distance of the translation stages with the caliper. After each target displacement was reached, we measured the total distance and repeated the experiment after bringing back the stage to the zero position (n = 10 independent measurements at each target position) with the electronic caliper. In both x- and y-directions, our analysis showed a high level of linearity and coefficients of determination (R^2^ = 0.9997 and 0.9998, respectively), proving that the motorized translation stages can reach the target displacement with high accuracy.

The Thorlabs servo motor controller and LabVIEW software are the hardware and software components that control the velocity of the motorized translation stage. A test was carried out to evaluate the system’s ability to accurately achieve the desired actuator velocity inputted in the LabView interface. This measurement was repeated (n = 10 independent measurements at each target actuator velocity) for different clamps velocities: 0.02, 0.1, 0.2, 1.0, 2.0, 3.0, 4.0, and 4.8 mm/s, in the x- and y-direction. After each set of tests, the mean actuator velocity was compared to its respective target velocity recorded using the digital position sensor of the linear stage. Our analysis shows that the translation stages are capable of maintaining the actuators velocity consistently in both directions, with 99.0 – 100% accuracy, and mean coefficients of determination ranging R^2^ = 0.9992–1.0. The experiments reported below were performed using an actuator velocity of 1.0 mm/s, for which we found an accuracy of 100% due to the fast feedback system of the linear stage.

### Actuation system: load cell

Measurement of the reaction force produced by a testing sample is achieved using sensitive miniature load cells (Model 31, Honeywell). We calibrated the load cell used in both axes of the system as follow: we used an L-bracket to position two standoffs and a dovetail optical rail vertically. The rail carrier is attached to the optical rail and the load cell is secured vertically at the end of the rail carrier. Weights (20 g − 500 g) were hung from the load cell to acquire a calibration curve between the output voltage of the load cell and the calibration weights. The slope of the calibration curve or scale factor was determined using a straight line curve fitting to the data. This test was repeated ten times, and the mean measured weight was compared to the actual (nominal) weight. Table 4 shows the mean measurement weight, the accuracy of the measurement (94.0–99.9%), and the coefficient of variance between each of the tests for each weight value (CV = 0–0.02).

**Table 4:** Accuracy and Coefficient of Variation of weight measurements acquired with the x- and y-direction load cells.

X-direction Load Cell			
Weight (g)	Measured Weight Mean ± SD (g)	Accuracy of Measurement (%)	Coefficient of Variation
0	0	–	0
20	19.85 ± 0.43	99.3%	0.02
50	50.10 ± 0.44	99.8%	0.009
100	100.01 ± 0.12	99.9%	0.001
200	200.13 ± 0.05	99.9%	0.0002
500	499.77 ± 0.54	99.5%	0.001
Y-direction Load Cell			
Weight (g)	Measured Weight Mean ± SD (g)	Accuracy of Measurement (%)	Coefficient of Variation
0	0	–	0
20	18.80 ± 0.27	94.0%	0.01
50	48.38 ± 0.24	96.8%	0.005
100	98.37 ± 0.26	98.4%	0.003
200	199.57 ± 0.46	99.8%	0.0023
500	501.35 ± 1.14	99.7%	0.0023

### Environment control: temperature

**Fig. 15:**
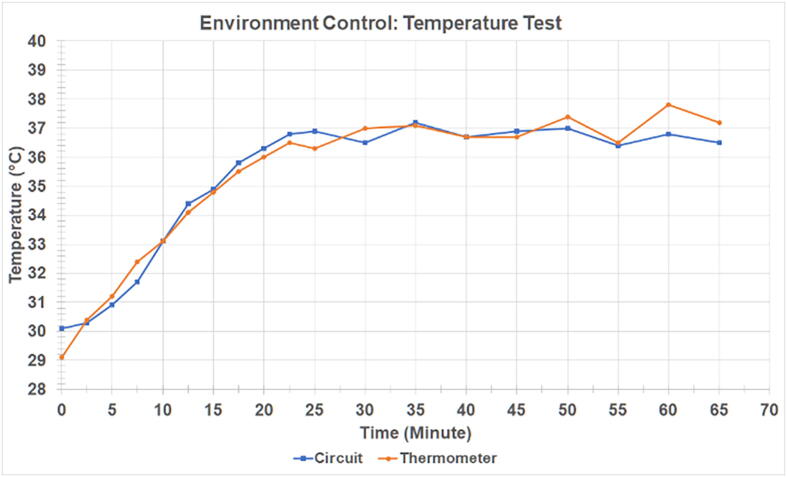
Dynamics of temperature. The temperature control system warms up the fluid in the testing chamber. The graph presents a comparison of temperature recorded from the temperature controller and the temperature measured with a thermocouple-based thermometer.

To obtain testing conditions similar to the physiological environment, the specimen chamber needs to maintain a constant temperature of the medium during the experiment. The validation test was designed to evaluate how long it would take for the water in the chamber to reach a temperature range of 37.0 ± 1.0 °C and sustain it for a long period of time. The readings of the W1209 circuit were compared to a standard digital thermometer for accuracy. Fig. 15 shows that the initial temperature of the water in the specimen chamber as per the thermometer readings was 29.1 °C, while the W1209 circuit read 30.1 °C. At the 25-minute mark, the temperature circuit reached 37.0 °C. After this point, the temperature was monitored for 40 min and the circuit was able to maintain a temperature range of 37.0 ± 1.0 °C. The thermometer’s lowest temperature reading was 36.5 °C and the highest temperature reading was 37.8 °C. It was surmised that the temperature circuit is capable of providing a warm environment for future studies.

### Gripping mechanism: slippage

The ability of the grips to prevent slippage of the sample was also evaluated. If the sample slips during the tensile test, the overall stress will decrease, either causing the recorded data to be skewed or prevent rupture. A test was designed where one sample was stretched in the x- and y-direction. A marked region (3.8 mm) of the sample was placed in the gripping mechanism and the samples were stretched 10 times for 10 mm. Each time, a caliper was used to measure any possible grip slippage as well as images were taken for digital measurement. After performing the evaluation test in the x- and y- directions 10 times, there was no noticeable or measurable slippage of the sample.

### Strain measurement: image analysis & strain mapping

**Fig. 16:**
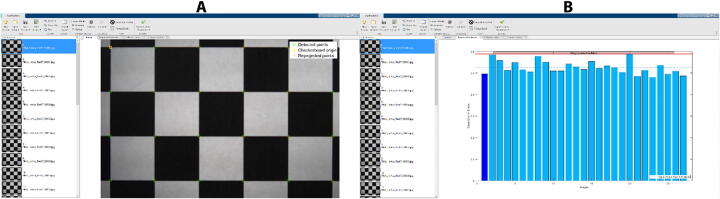
Camera Calibration and accuracy measurement. (A) The original checkerboard image (1.0 cm × 1.0 cm) with the corners detected (green circles) and the reprojected points (red crosshair markers). (B) Histogram of mean error in pixels for each analyzed image, showing an overall mean error of 0.53 pixels. (For interpretation of the references to color in this figure legend, the reader is referred to the web version of this article.)

The digital camera was calibrated using the Computer Vision Toolbox and the cameraCalibrator app in Matlab (V. 2021b, Mathworks). A 10x10 checkerboard pattern image was created comprising black and white 10 mm × 10 mm squares with 1200dpi resolution. The checkerboard pattern was printed using a high resolution printer (ColorLaserJet Pro MFP M476dw, Hewlett Packard) and placed in a flat surface at the focal distance of the Tamron M111FM25 lens. Ten images were acquired by the system and loaded into the cameraCalibrator app in Matlab. The camera field of view (5472 × 3648 pixels) comprises at least 4 by 6 squares of the checkerboard pattern. The cameraCalibrator app detects the checkerboard corners in the images and generates the coordinate system in millimeters (Fig. 16**A**). The camera parameters (accuracy, skew and lens distortion errors) were calculated for each image based on the position of the checkerboard corners within the coordinate system and estimate the reprojection errors in pixels. The bar graph (Fig. 16**B**) indicates that the accuracy of the calibration is 0.53 pixels, where each bar represents the mean reprojection error for the corresponding calibration image. The reprojection errors are the distances between the corner points detected in the image, and the corresponding ideal points projected into the image. The estimated skew error is 3.63 pixels, the radial distortion coefficients are *k_1_* = 0.036 and *k_2_* = 62.847, and the tangential distortion coefficients of the lens are *k_1_* = 0.003 and *k_2_* = 0.002, where $x_{\mathrm{distorted}}=x\left(1+k_{1}r^{2}+k_{2}r^{4}\right),y_{\mathrm{distorted}}=y\left(1+k_{1}r^{2}+k_{2}r^{4}\right)$, *x* and *y* are the undistorted pixel locations and $r^{2}=x^{2}+y^{2}$. The measured camera parameters obtained by the cameraCalibrator app are used for correcting the measured distortion and produce distortion-free images.

The quality of the DIC analysis largely relies on the speckles pattern of the sample being analyzed. To obtain accurate and reliable data, we followed the recommendations for good DIC testing [17]. First, the paint used for the pattern should be of high contrast against the samples’ color. Since the material tested in this study is transparent, we used a water-based black ink. Then, the speckles size should be in the range of 3–5 pixels and the pattern density between 20 and 50% of the sample’s area. We used a professional airbrushing paint system (VIVOHOME, California, USA) to ensure a constant speckle size and a uniform pattern. The spraying technique was optimized using the pattern quality assessment available in GOM Correlate.

Once images are imported into GOM, the user selects the size of squared facets and their respective overlapping area to be used for further calculations. To ensure accurate results, the facet should contain at least three pattern points so that one subset can be distinguished from all other subsets in the ROI. In this study, we used a facet size of 17x17 pixels and a point distance of 15 pixels, which gave the best outcome in terms of pattern quality.

### Uniaxial and biaxial tensile tests

**Fig. 17:**
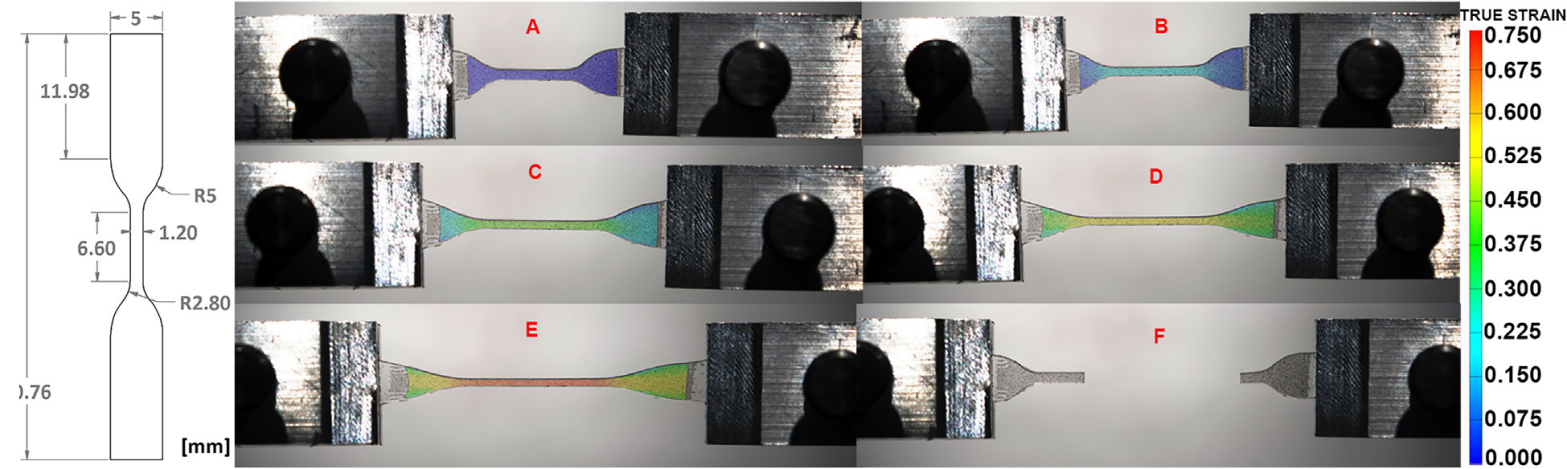
(Left) Technical drawing of the uniaxial sample, from the standard ASTM D412-Type C. (Right) Strain map of a uniaxial sample of Sylgard184 (n = 10) calculated in GOM Correlate in empty bath. (A) Sample is in its underformed state. (B-E) Sample is progressively stretched and the increase in strain is mapped on its surface. (F) Sample has ruptured in the gauge region, where strain is the highest.

To evaluate our tensile test protocol, we tested ten uniaxial samples in the axial direction, and ten biaxial samples in both x- and y-directions until rupture. The polymer mixture that was used for preparing the testing samples is Polydimethylsiloxane (PDMS, or Sylgard 184, Dow Corning). Sylgard 184 [18], [19], [20], [21] is a widely used elastomer in the scientific community due to its biocompatibility, optical transparency, flexibility and mechanical properties. Given its ability to change elasticity depending on the base to curing agent ratio, Sylgard184 has been often considered as a laboratory model material for different soft tissues [22], [23], [24]. Samples were prepared with a base to curing agent ratio of 10:1, cured at a temperature of 100 °C and then sprayed to create the speckle pattern needed for DIC. For uniaxial tests, dumbbell-shape samples was scaled-down to 20% of original ASTM D412 standard sample size for tensile testing of rubbers and elastomers (Fig. 17), with a thickness of 0.6 mm. For biaxial tests, cruciform samples were derived from the uniaxial geometry, maintaining the same dimensions (Fig. 20**A**).

The experimental procedure consisted of applying 10-cylces preconditioning stretch at 10% strain, followed by one-single pull to rupture. Fig. 17 demonstrates the progression of the strain in the ROI of one sample from the undeformed state until failure. Fig. 17**A** shows the sample in the unloaded configuration. As the translation stages move to the target displacement, the program is able to map the strain profile of the material. In Fig. 17**E**, the gauge region has reached the ultimate strain of polymer mixture and in Fig. 17**F**, the sample has ruptured, and the strain map ends.

**Fig. 18:**
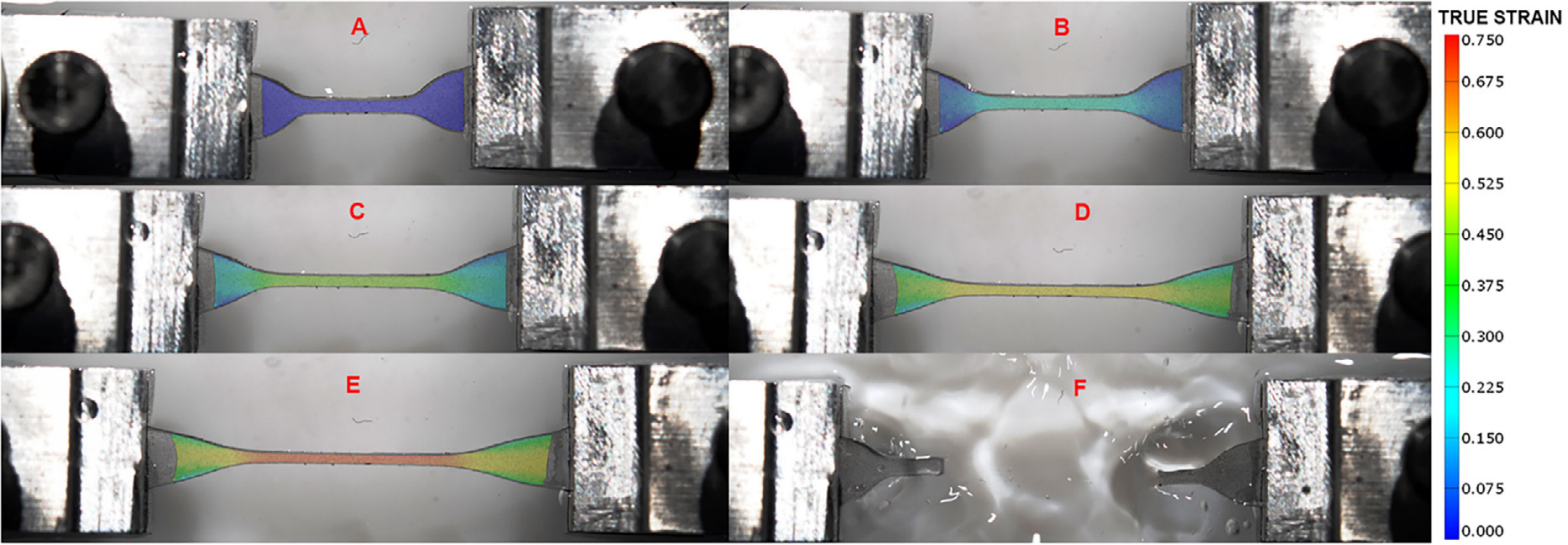
Strain map of a uniaxial sample of Sylgard184 calculated in GOM Correlate in bath filled with water. (A) Sample is in its underformed state. (B-E) Sample is progressively stretched and the increase in strain is mapped on its surface. (F) Sample has ruptured in the gauge region, where strain is the highest, causing fluid perturbation on its surrounding.

When studying soft tissues, the sample should ideally be maintained in a bath of physiological solution. To validate the DIC analysis when testing samples in solution, we also performed experiments where our bath was filled with water until wetting the sample and keeping it humid throughout the test. In this case, we sprayed the samples using black acrylic paint, so that the pattern wasn’t washed away by the fluid. The strain mapping until rupture is reported in Fig. 18**.** The software was able to calculate the strain profile for all stretch levels and the ultimate strain reported was the same as for samples tested on the bath without water. These results validate the strain mapping protocol when using our device whether the samples can be kept dry or need to be tested in physiological solution.

**Fig. 19:**
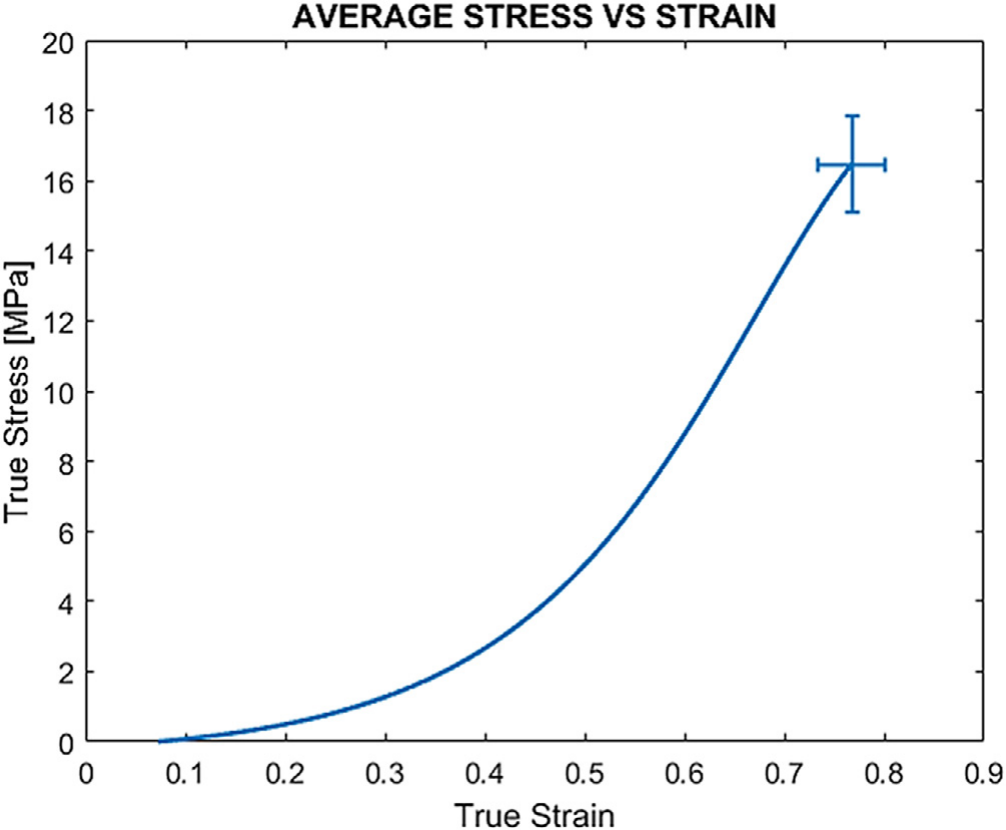
Average true stress–strain curve for Sylgard 184 (n = 10), calculated with DIC analysis. Error bars represent ± SD of ultimate stress and strain.

The true stress values were calculated by deriving the reduction in cross-sectional area from the change in width in the gauge region throughout the test, assuming the material as isotropic. The individual and average true stress and strain curves were obtained combining the data from the tensile system and the DIC analysis in a custom-made MATLAB script. The average response of the material under uniaxial tension is reported in Fig. 19, which shows the typical hyperelasticity of Sylgard 184. Our results agree with data previously reported in the literature for Sylgard 184 [25], [26], [27], demonstrating the reliability of our system.

**Fig. 20:**
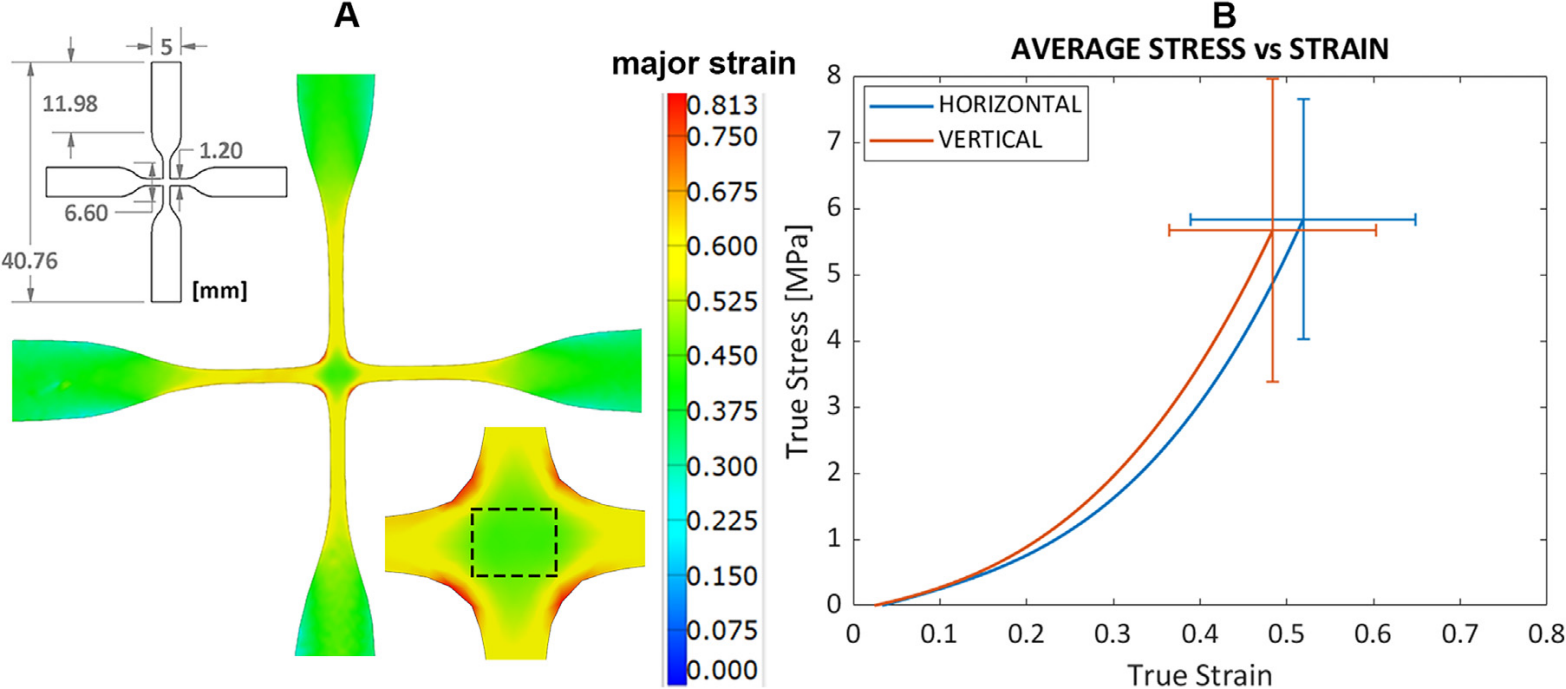
Testing of biaxial sample of Sylgard184 (n = 10). (A) Technical drawing of the cruciform sample; Two-dimensional mapping of the true major strain of one sample from GOM, with a closer view of the test region, showing the uniform strain field in the central area. (B) Average true stress–strain curves for the two tensile directions.

The results for biaxial tests are reported in Fig. 20*.* For this case, since the material exhibits the highest engineering stresses measured by DIC at the central corners (Fig. 20**A**) and always failed at that location, it was not possible to derive the change in cross-section area in this region. Thus, to calculate the true stresses, we first derived the engineering strain from the true strain values obtained in GOM and then computed the Cauchy stresses for each sample in the two directions. The strain data were extracted from the test region where the strain field is uniform. The average true stress and strain curves in the longitudinal (horizontal) and vertical direction are reported in Fig. 20**B** and show no significant difference between the two directions of loading. Our results show the ability of our system to perform and record accurate data for a biaxial tensile test. The material exhibits a mechanical response that is very close in the horizontal and vertical direction, confirming its isotropic nature.

## CRediT authorship contribution statement

**Andrea Corti:** Conceptualization, Data curation, Validation, Writing – review & editing. **Tariq Shameen:** Conceptualization, Data curation, Validation, Writing – original draft. **Shivang Sharma:** Data curation, Methodology. **Annalisa De Paolis:** Data curation, Methodology. **Luis Cardoso:** Conceptualization, Supervision, Writing – review & editing, Funding acquisition.

## Declaration of Competing Interest

The authors declare that they have no known competing financial interests or personal relationships that could have appeared to influence the work reported in this paper.

## References

[1] Azadani A.N. (2012). Comparison of mechanical properties of human ascending aorta and aortic sinuses. Ann. Thorac. Surg..

[2] Benedik J. (2014). Comparison of ascending aortic cohesion between patients with bicuspid aortic valve stenosis and regurgitation. Eur. J. Cardiothorac. Surg..

[3] Fehervary H. (2016). Planar biaxial testing of soft biological tissue using rakes: A critical analysis of protocol and fitting process. J. Mech. Behav. Biomed. Mater..

[4] Fehervary H. (2018). How important is sample alignment in planar biaxial testing of anisotropic soft biological tissues? A finite element study. J. Mech. Behav. Biomed. Mater..

[5] Fata B. (2013). Regional structural and biomechanical alterations of the ovine main pulmonary artery during postnatal growth. J. Biomech. Eng..

[6] Imsirovic J. (2015). Design of a Novel Equi-Biaxial Stretcher for Live Cellular and Subcellular Imaging. PLoS ONE.

[7] Li L. (2019). A miniaturized biaxial tensile apparatus based on torsional loading design. Rev. Sci. Instrum..

[8] Potter S. (2018). A Novel Small-Specimen Planar Biaxial Testing System With Full In-Plane Deformation Control. J. Biomech. Eng..

[9] Faturechi R., Hashemi A., Abolfathi N. (2014). A tensile machine with a novel optical load cell for soft biological tissues application. J. Med. Eng. Technol..

[10] Sang C., Maiti S., Fortunato R.N., Kofler J., Robertson A.M. (2018). A Uniaxial Testing Approach for Consistent Failure in Vascular Tissues. J. Biomech. Eng..

[11] Jiang M. (2021). A versatile biaxial testing platform for soft tissues. J. Mech. Behav. Biomed. Mater..

[12] King J.D., York S.L., Saunders M.M. (2016). Design, fabrication and characterization of a pure uniaxial microloading system for biologic testing. Med. Eng. Phys..

[13] Shiwarski D.J. (2020). 3D printed biaxial stretcher compatible with live fluorescence microscopy. HardwareX.

[14] Jiang M. (2020). Clamping soft biologic tissues for uniaxial tensile testing: A brief survey of current methods and development of a novel clamping mechanism. J. Mech. Behav. Biomed. Mater..

[15] Bell B.J., Nauman E., Voytik-Harbin S.L. (2012). Multiscale strain analysis of tissue equivalents using a custom-designed biaxial testing device. Biophys. J ..

[16] Deplano V. (2016). Biaxial tensile tests of the porcine ascending aorta. J. Biomech..

[17] Jones E.M.C. (2018). *A Good Practices Guide for Digital Image Correlation*, in *International Digital Image Correlation*. Society..

[18] Johnston I.D. (2014). Mechanical characterization of bulk Sylgard 184 for microfluidics and microengineering. J. Micromechanics and Microengineering.

[19] Mata A., Fleischman A.J., Roy S. (2005). Characterization of polydimethylsiloxane (PDMS) properties for biomedical micro/nanosystems. Biomed. Microdevices.

[20] Wu C.L. (2009). Static and dynamic mechanical properties of polydimethylsiloxane/carbon nanotube nanocomposites. Thin Solid Films.

[21] Schneider F. (2008). Mechanical properties of silicones for MEMS. J. Micromechanics Microengineering.

[22] Ramesh R. (2008). Comparison of radial expansion of stents within mock vessels molded with a target bent radius versus straight mock vessels bent to a target radius. Biomed. Sci. Instrum..

[23] Hecker L. (2005). Development of a three-dimensional physiological model of the internal anal sphincter bioengineered in vitro from isolated smooth muscle cells. Am. J. Physiol. Gastrointest. Liver Physiol..

[24] Colombo A. (2010). A method to develop mock arteries suitable for cell seeding and in-vitro cell culture experiments. J. Mech. Behav. Biomed. Mater..

[25] Liu M., Chen Q. (2007). Characterization study of bonded and unbonded polydimethylsiloxane aimed for bio-micro-electromechanical systems-related applications. J. Micro/Nanolithogr. MEMS MOEMS.

[26] Liu M., Sun J., Chen Q. (2009). Influences of heating temperature on mechanical properties of polydimethylsiloxane. Sens. Actuators, A.

[27] Liu M. (2009). Thickness-dependent mechanical properties of polydimethylsiloxane membranes. J. Micromech. Microeng..

